# Bio‐Inspired Interlocking Micro‐Patterning for Tunable, Switchable and Selective Adhesion in Wet and Dusty Environments

**DOI:** 10.1002/smll.202410527

**Published:** 2025-02-26

**Authors:** Marco Bruno, Luigi Portaluri, Massimo De Vittorio, Stanislav Gorb, Michele Scaraggi

**Affiliations:** ^1^ University of Salento Department of Engineering for Innovation Via per Monteroni Lecce (LE) 73100 Italy; ^2^ Italian Institute of Technology Center for Biomolecular Nanotechnologies Via Eugenio Barsanti, 14 Arnesano (LE) 73010 Italy; ^3^ Kiel University, Zoological Institute Department of Functional Morphology and Biomechanics Am Botanischen Garten 1-9 D 24118 Kiel Germany

**Keywords:** adhesion in dust, bio‐inspired adhesion, mechanical adhesion, pattern grasping, underwater adhesion

## Abstract

Achieving adhesion under unfavorable conditions, such as when van der Waals interaction is not available or in dust environments, is crucial in applications ranging from surgical sutures to wound‐healing tapes, underwater adhesives, robotic grippers, and space grasping. Interestingly, plants, animals, and microorganisms living in such environmental conditions show surface morphological traits optimized to achieve mechanical interlocking. Thus, they achieve an effective work of adhesion thanks to the interplay of friction and interfacially‐storable elastic energy, which otherwise typically suppress adhesion. In this work, the design and fabrication fundamentals for achieving tunable, switchable, and robust mechanical adhesion is provided under a general environmental condition, such as wet or dusty, bio‐mimicking natural solutions. A theoretical framework for the design of mechanical adhesion, based on mean‐field continuum contact mechanics, is suggested and validated experimentally. This study can pave the way for the development of new technologies to be employed in situations where conventional adhesives may be ineffective, such as for surfaces exposed to water, solvent vapors, lubricants, high temperatures, dusty environments, high vacuum, or aerospace applications, or processes where switching and selective adhesion is needed such as grasping and sorting applications in the semiconductor industry.

## Graphical abstract

A new class of microfastener geometries is proposed, coming from biomimetic considerations and design optimization based on an original theoretical framework that is validated through testing and simulations. Demonstrators are assembled to present the impact that this technology can have on multiple fields: From precision grasping to drug delivery applications and underwater adhesives.

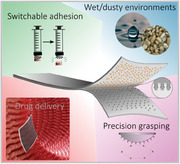

## 1 Introduction

Achieving adhesion can be challenging when intermolecular forces, such as van der Waals interactions, are unavailable or in dusty/wet environments. The absence of van der Walls interactions is typically encountered in a number of applications, including surgical sutures, wound‐healing tapes, underwater adhesives, robotic grippers, and space grasping. Plants, animals, and microorganisms living in similar environments exhibit surface morphological traits optimized for mechanical interlocking.^[^ ^1^, ^2^, ^3^ ^]^ In particular, they increase the effective work of adhesion through the interplay of friction and interfacially‐stored elastic energy, the latter otherwise typically suppressing the adhesion at the macroscale.^[^ ^4^ ^]^

Selective and switchable adhesion is, therefore, a fundamental adaptation capability in nature.^[^ ^5^ ^]^ Furthermore, reversible attachments are present everywhere, and we use everyday tools that rely on these mechanisms.^[^ ^6^ ^]^ Design of bioinspired adhesives typically relies on the adoption of the well‐known contact splitting, achieved through the microfabrication of a variety of surface microtextures, such as needles,^[^ ^7^, ^8^, ^9^ ^]^ pillars,^[^ ^10^, ^11^, ^12^, ^13^ ^]^ spherical/elliptical^[^ ^14^, ^15^ ^]^ micro geometries, mushroom‐shaped pillars,^[^ ^16^, ^17^, ^18^ ^]^ hook‐loop interfaces.^[^ ^19^, ^20^ ^]^ Switchable adhesion can also be achieved by adopting needle structures that penetrate a soft substrate and inflate afterward^[^ ^21^ ^]^ or by forming wrinkles on the surface.^[^ ^22^ ^]^ It can also be achieved by orienting needles that penetrate inside a soft substrate and can withstand tangential loading in their direction of orientation.^[^ ^9^, ^23^ ^]^

Reversible adhesives have already been developed in a variety of designs, each with a different approach to control adhesion. Dry adhesives based on van der Waals forces ^[^ ^24^, ^25^, ^26^, ^27^, ^28^ ^]^ can switch adhesion by changing the morphology of a surface. Suction‐based mechanisms, on the other hand, rely on a pressure differential to achieve controlled adhesion. The latter are commonly proposed for underwater applications, taking inspiration from the octopus suction cups.^[^ ^29^, ^30^ ^]^ Each of these approaches is characterized by strengths and limitations. For instance, while dry adhesion, mediated by van der Waals forces, is effective on clean, dry surfaces, it is highly sensitive to contamination and humidity. Additionally, suction‐based mechanisms require continuous actuation and specific contact conditions and can also show a limited load‐bearing capacity when subjected to shear forces. In contrast, mechanical interlocking mechanisms can behave both as active or passive adhesives. As elucidated in the following, their adhesive performances are not affected by surface contamination, and their load capacity can be tailored by varying the stiffness of the interlockers.

When focusing on mechanical adhesion, we note that whilst the limitation of interlocking intrinsically lies in the surface selectivity requiring both surfaces to be specifically engineered, this characteristic enables a selective attachment on specific surfaces.^[^ ^31^ ^]^ Some insects and reptiles use this strategy to stabilize and clamp parts of their bodies temporarily or permanently.^[^ ^15^, ^32^ ^]^ In these natural interlocking mechanisms, hooks or needles are the typical micro geometry, as can be observed in the way parasites attach to their hosts,^[^ ^21^ ^]^ or in plant burrs that can be dispersed through animal fur.^[^ ^1^, ^33^ ^]^ Depending on the geometry, interlocking can reach attachment strength near the tensile strength of the material,^[^ ^33^, ^34^ ^]^ especially in tangential loading.^[^ ^12^ ^]^ Predicting the role of dimensions, shape, and distribution of surface outgrowths in natural systems is crucial for understanding their mechanical behavior in contact. An analytical frictional model of two single parabolic pins in sliding contact has been presented in ref. [35] and the numerical implementation of their collective behavior in ref. [36]. However, no model provides a comprehensive understanding of the influence of more general micro geometries and their single‐paired and collective (statistical) contact mechanics.

Mechanical adhesion also provides a viable alternative to chemical bonding in wet environments.^[^ ^37^ ^]^ Developing water‐resistant adhesives for these conditions is challenging^[^ ^38^, ^39^, ^40^ ^]^ due to the formation of a hydration layer on submerged surfaces, which impedes chemical adhesion^[^ ^41^ ^]^ Similar complexities apply in the case of environments with solvents, vapors, lubricants, or dust. Indeed, hook‐and‐loop fasteners (or interlockers) and needle interlockers have been successfully applied in space applications^[^ ^42^ ^]^ to enable easily reversible attachments. Interlocking systems also show promise for medical applications, such as adhering soft patches to the complex, mucus‐covered surfaces of the gastrointestinal tract.^[^ ^43^ ^]^

As soft materials become increasingly prevalent in fields such as wearable electronics, soft robotics, and electronic skins,^[^ ^44^ ^]^ interlocking emerges as a reliable method for achieving durable adhesion. Moreover, high temperatures and dusty environments pose significant challenges for conventional adhesives, often leading to performance degradation or even failure. In contrast, interlocking structures can be engineered to operate in any of these conditions.^[^ ^45^ ^]^ Several studies were undertaken to build artificial systems to realistically describe the behavior of co‐opted natural fields of probabilistic fasteners in contact and to make further steps into developing engineering applications using this very promising kind of general‐purpose bonding technology.^[^ ^32^, ^46^, ^47^, ^48^, ^49^ ^]^

A number of studies investigate mechanical adhesion from a theoretical standpoint. However, each is limited to a specific shape and mode of interlocking.^[^ ^2^, ^40^, ^50^ ^]^ Unfortunately, this approach does not provide a fundamental understanding of the interlocking phenomena in terms of interaction statistics and the mechanics at the scale of the single contact. Indeed, coupling the local contact mechanics (say, at the scale of the micro geometry) with the whole‐scale deformation of a single‐pair interlocking structure is a theoretically difficult task. This task is typically simplified by assuming that only a portion of the single structure deforms, while the rest is perfectly rigid,^[^ ^16^, ^51^, ^52^ ^]^ or considering frictionless and non‐adhesive contact.^[^ ^53^, ^54^ ^]^

In some cases, both friction and van der Waals forces are considered, but for highly‐simplified, high aspect‐ratio geometries such as beam‐like elements.^[^ ^43^, ^47^ ^]^ Furthermore, fundamental studies on the role of the distribution of the interlocking structures on mechanical adhesion are very limited.^[^ ^55^ ^]^ Oftentimes, the number of interacting structures is typically oversimplified as the characteristic number of the lattice arrangement or described by assuming their probability distribution function described with semi‐empirical relations.^[^ ^56^ ^]^ This overlap of single‐pair and statistical contact mechanics approximations can easily lead to inaccurate results and hinder the possibility of designing application‐specific interlockers with optimized distribution and micro‐geometry.

This study is devoted to overtaking the aforementioned limitations by presenting a general design and fabrication approach for creating tunable, switchable, and robust mechanical adhesion that is effective under general environmental conditions, including wet and dusty ones mimicking natural solutions. We propose a comprehensive theoretical framework for modeling mechanical adhesion based on mean‐field continuum contact mechanics, along with a facile micro‐fabrication and testing process of the patterned surfaces. In particular, the contact mechanics theory for a single interlocking interaction is first derived and subsequently adopted within the homogenized macroscale contact problem, allowing us to achieve a general understanding of the involved mechanisms and to determine the effective work of adhesion. Furthermore, the single scale and mean field models are compared with the experimental findings. Upon the adoption of optimized micro‐geometries, in‐house‐built demonstrators will be shown to demonstrate the capabilities of interlocking surfaces in selected technological applications. This research can pave the way for the design and fabrication of new technologies to be adopted when conventional adhesives fail, such as on surfaces exposed to water, solvent vapors, lubricants, high temperatures, dusty environments, high vacuum, or in space applications.^[^ ^37^, ^57^, ^58^ ^]^ These technologies can also be beneficial in processes requiring switching and selective adhesion, such as grasping and sorting in the semiconductor industry and patches for selective drug release.

## 2 Results and Discussion

### 2.1 Interlocking Micro‐structural Geometries: Inspiration from Nature

In insects, microstructure‐covered pairs of surfaces often function for attachment between different body parts^[^ ^35^, ^59^, ^60^, ^61^, ^62^, ^63^ ^]^ These systems, showing mechanical adhesion, are often called probabilistic fasteners since the size, shape and density of the microstructures on both surfaces do not correspond exactly to each other, but rather generate a kind of co‐opted fields. Thus, mechanical interactions between the micro‐outgrowths of both surfaces occur without precise positioning of both surfaces.^[^ ^59^ ^]^ When the fields of the micro‐outgrowths are in contact, they also prevent the sliding of surfaces. Mechanical adhesion or attachment in the pull‐off direction, in this case, must depend on the size, shape, distribution density, and material properties of outgrowths (also called “elements”) and is fast, precise, and reversible. Individual elements do not necessarily have to be hook‐shaped as in the case of classical Velcro fasteners. Moreover, the mechanism of attachment in such systems is definitely different from the typical hook‐and‐loop Velcro™ principle but has been only partially experimentally studied for the case of probabilistic fasteners with parabolic contact elements.^[^ ^35^ ^]^ This kind of system has been previously described in a variety of insects at different body parts: Unguitractor plate,^[^ ^64^ ^]^ intersegmental fixators of leg joints,^[^ ^65^ ^]^ synchronizing mechanisms of contralateral legs in cicada,^[^ ^66^ ^]^ dragonfly head‐arresting system,^[^ ^31^ ^]^ and especially in wing‐attachment devices.^[^ ^60^, ^61^, ^67^, ^68^ ^]^ The most significant similarity of these devices in different organisms is that co‐opted fields of cuticular outgrowths are present on two different body parts. Sometimes, as in the case of the forewing fixators of beetles, more than just one pair (up to eight pairs) of co‐opted structures are involved to provide the proper kinematics to the probabilistic mechanical joint. In such cases, each pair favors the movement of the closed wings in one preferred direction. The head arrester of adult dragonflies, instead, immobilizes the head in some critical moments when it may experience strong mechanical disturbances.^[^ ^31^ ^]^

**Figure 1:**
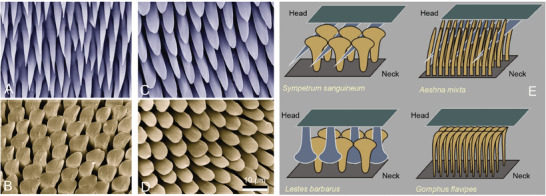
Surface microstructures of the head‐arresting contact system: The rear surface of the head and neck sclerites. A,B) Scanning electron microscopy images in the dragonfly *Idionyx saffronata* (Libellulidae): Head (A) and neck (B). C,D) Scanning electron microscopy images in the damselfly *Perissolestes romulus* (Perilestidae): Head (C) and neck (D). The scale bar in D also applies to A, B, and C. E) Schematic representation of interlocking of microstructures on corresponding fields of the head‐arresting contact system in three species of dragonflies, *Sympetrum sanguineum* (Libellulidae), *Aeshna mixta* (Aeshnidae), *Gomphus flavipe*s (Gomphidae) and one species of damselflies, *Lestes barbarus* (Lestidae).

Different shapes of outgrowths in functionally corresponding fields have been previously described,^[^ ^61^ ^]^ as shown in **Figure** **1**: i) Hook‐like or cone‐shaped or parabolic elements and mushroom‐like elements, ii) cone‐shaped or parabolic elements on both surfaces, iii) clavate elements on both surfaces and iv) plate‐like elements on both surfaces.^[^ ^35^ ^]^ Surface outgrowths of probabilistic fasteners vary in different biological systems and even between corresponding fields within the same system. However, in the pair of corresponding surfaces, one could expect some constraints in the size and distribution of co‐opted areas of outgrowths. In the dragonfly head arrester, the size of the outgrowths is usually larger in large species, and their density is lower, but the density of outgrowths on co‐opted fields of one species is surprisingly never the same. A similar observation has been made for the co‐opted microtrichia fields of the forewing fixators of the beetles.^[^ ^60^ ^]^ In the example of the dragonfly arrester, the density is higher on the neck sclerite than on the rear surface of the head.^[^ ^65^ ^]^ It is even more difficult to explain the difference in the outgrowth density on the corresponding structures due to the lack of a fundamental theory of probabilistic fastening. In addition, the distribution of the outgrowths in the biological systems shows a roughly hexagonal pattern, which is generally accepted to represent the highest package density of structures, but this pattern is far from an ideal one. It remains unknown whether these features have any significance on the function of co‐opted fields. However, recent numerical simulation shows that such mismatches lead to an increase in the resulting pull‐off force.^[^ ^36^ ^]^

Interlocking systems also appear in other species, acting as a biomechanical constraint. They are fundamental in the correct development of the skull for humans,^[^ ^69^ ^]^ since the interlocking of the flat bones of the skull both guarantees the protection of the brain with secure attachments and the relative movement of the bones during growth. Teeth also exploit the same principle of interlocking to firmly attach to the mandible and maxilla bones, both adapting to the bone changes during growth^[^ ^70^ ^]^ and withstanding frequent and intense mechanical stresses.

### 2.2 Theory of Mechanical Adhesion

**Figure 2:**
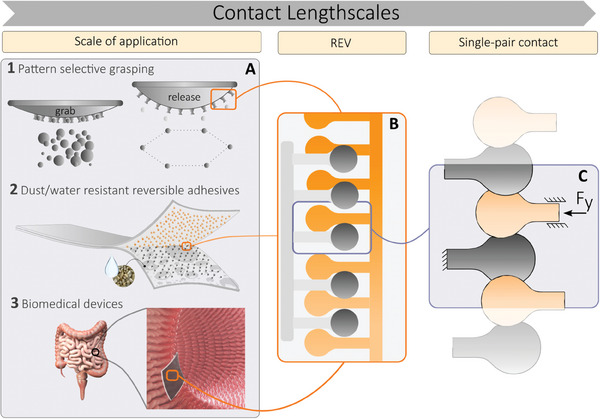
Schematic representation of representative mechanical adhesion applications and their design approach based on our multi‐scale contact mechanics. A) Application lengthscale, where adhesion is detected as an emergent behavior: A1) Tools for automatic sorting, grasping, and pattern arranging; A2) Reversible adhesive patches for dusty and wet environments; A3) Patient‐ and tissue‐specific adhesive patches for drug release, such as adhering to the intestinal tract. B) Representative elementary volume of the interface (REV), much smaller than the size of the macro‐contact, however much larger than the single microstructure to enable the evaluation of the statistical (collective) behavior of the micro‐contacts. C) In this work, the smallest scale of the interaction is characterized by the single‐pair contact.

The theory is developed within a multi‐scale architecture, characterized by three lengthscales as shown in **Figure** **2**. The application lengthscale is the largest scale of the interaction, where the interlocking can be detected as an emerging behavior, as shown in Figure 2A illustrating 1) pattern‐selective grasping, 2) dust/water resistant adhesion, and 3) tissue‐specific patches. Upon magnifying the contact zone, the representative elementary volume (REV) of the interface can be identified, as seen in Figure 2B, where an ensemble of micro‐fasteners is statistically operating under the same locally‐averaged interface separation. By further increasing the magnification, the single interaction pair is identified and modeled with a deterministic contact theory, as observed in Figure 2C.

An averaging approach is adopted to link the single interaction behavior (Figure 2C) to the collective behavior at the application scale (Figure 2A) as follows: Starting from the single‐pair contact mechanics (derived in Section S4, Supporting Information), the contact in the REV is assumed to occur between independent single‐pairs under a constant separation (see the nominally parallel surfaces in Figure 2B), whose placement statistics depends on the specific micropattern fabrication process. In particular, the probability density function of the distance between microstructures in their non‐deformed state (*p*(*I*_0_)) and in the REV domain is determined, as well as the expected number of mating microstructures per unit area at a given distance from each other (Section S5, Supporting Information). Finally, the equation of state (or Cohesive Zone Model, CZM), linking the locally‐averaged contact pressure with the interface separation, is determined for the specific REV, together with the effective work of mechanical adhesion Δγ_eff_ (Section S6, Supporting Information). The CZM homogenizes the nature of the interaction of single‐pairs as well as the statistics of their lattice positioning. This model can then be implemented as an effective interaction law and coupled with a smooth formulation (microstructures‐free) of the contact mechanics at the application scale, as typically done in randomly rough contact mechanics.^[^ ^71^ ^]^ It is worth noting that this approach relies on the assumption that single interlocking structures are significantly smaller than the application lengthscale. Hence this model will predict more accurately the adhesion performance of the same surfaces whe patterned with smaller interlockers.

Furthermore, the CZM allows us to determine the attachment (or engagement) pressure σ_a_ and the pull‐off (or disengagement) pressure σ_d_, which are the maximum compressive and detachment traction applied at the representative elementary volume of the interface, respectively. These parameters are extremely important performance indicators to be evaluated for the specific application of mechanical adhesion. We stress that increasing the local friction can greatly enhance the pull‐off pressure σ_d_ needed to disengage the locking mechanism. In contrast, the effective work of adhesion is null for frictionless contacts.

#### 2.2.1 Single‐Pair Contact

**Figure 3:**
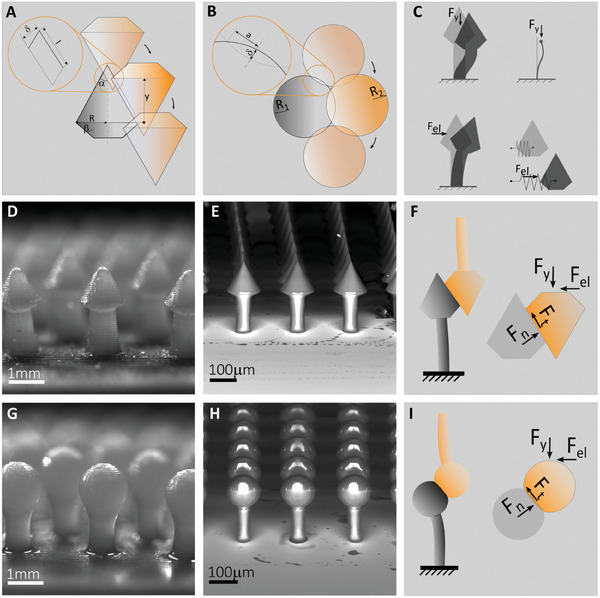
Microscope acquisitions of the fabricated interlockers and schematics of their single‐pair interaction, with identification of the contact parameters. A) Interlocking kinematics for the double conical micro geometry. Here, δ is the contact penetration, *l* is the contact length, *y* is the out‐of‐plane distance between the bases of the cones. B) Interlocking kinematics for the spherical micro geometry. Here, *a* is the contact radius, *y* is the out‐of‐plane distance between the centers. C)‐top, Sticking‐induced buckling upon contact. C)‐bottom, The in‐plane (bending) micro geometry deformation equivalent stiffness. D) Optical microscope image of a 3D printed patterned dome with double conical features (R=0.7mm, α=π/6, β=π/3); the material is polymer‐B (polymer‐B is defined in the Materials Section in Experimental Section). E) Scanning electron microscope image of a glass slide patterned with double conical features (R=70µ*m*, α=π/6, β=π/3) fabricated through two photo‐polymerization technique using Nanoscribe IP‐S photoresin. F) Free body diagram of the double conical interlocker during the interaction with its mating counterpart, *F*_y_, *F*_el_, *F*_n_, *F*_t_ are, respectively, the out‐of‐plane, in‐plane, normal‐ and tangential‐contact forces. G) Optical microscope image of a 3D printed patterned dome with spherical features (R=0.7mm), made of polymer‐B material. H) Scanning electron microscope image of a glass slide patterned with spherical features (R=70 µm), fabricated through two‐photon polymerization technique using Nanoscribe IP‐S photoresin. I) Free body diagram of the spherical interlocker during the interaction with its mating counterpart, *F*_y_, *F*_el_, *F*_n_, *F*_t_ are, respectively, the out‐of‐plane and in‐plane forces, and the normal and tangential contact forces.

The generic single‐pair interaction is shown in the schematic of **Figure** **3**, with the indication of two representative micro geometries adopted in this study (Figure 3A,B), and related deformation modes of the supporting rod (Figure 3C). A list of symbols used throughout this work is provided at the beginning of the Supporting Information.

The contact, assumed quasi‐static, is based on the Hertzian contact mechanics for the spherical geometry and a simplified contact model with a Winkler foundation for the conical geometry,^[^ ^72^, ^73^ ^]^ coupled with a Da Vinci‐Coulomb local friction. More complex micro geometries would require local contact mechanics to be handled numerically, such as with finite element methods; however, this does not affect the multiscale procedure developed in this work. Hereinafter, the local adhesion is not included in the model since it does not quantitatively affect the overall effective work of adhesion (see the discussion in Section S4.1, Supporting Information).

*Spherical interaction*

**Figure 4:**
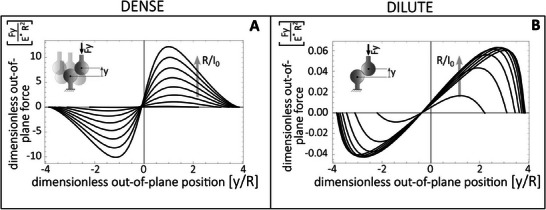
Normalized out‐of‐plane force during the single‐pair interaction of spherical interlocking structures. A) Out‐of‐plane force during approaching motion of densely packed spheres at various *R*/*I*_0_ values. B) Out‐of‐plane force during approaching motion of non‐densely (i.e. dilute) packed spheres at various *R*/*I*_0_ values. *R*/*I*_0_ varies from 0.4 to 2, with a step of 0.2. The coefficient of friction μ is 0.25 (as for wet environment), E* is the equivalent Young modulus (4MPa).

As discussed earlier, spheres (on the top of elastic rods) are interlocker tips also adopted in natural probabilistic fasteners. Here, the same tip is considered due to the simple geometrical description; its single‐pair contact model is derived in Sections S4.1 (Supporting Information). This geometry also helps to shed light on the sticking‐induced buckling phenomenon. In particular, the interaction of two smoothly curved (derivative‐continuous) tips shows a contacting regime characterized by a sticking frictional behavior when their in‐plane distance *I* is below a critical friction‐dependent threshold *I*_crit_. This critical length decreases as friction increases. For spheres $I_{\mathrm{crit}}=2R\mu /\sqrt{1+\mu^{2}}$, as derived in Section 4.1.3 (Supporting Information). As expected, for ideal infinite friction, the critical distance is twice the sphere radius, i.e. any contact between tips occurs under stick interaction. In a sticking contact, increasing the externally applied normal force might induce either the elastic buckling of the supporting rods or the failure of the patterned surface. However, no interlocking will occur for in‐plane distances below *I*_crit_. In **Figure** **4**, the out‐of‐plane force *F*_y_ is shown for the spherical tip, under approaching motion. For receding motion, the resulting curves are mirrored along the reference axes due to the contact symmetry. In particular, both the dense (i.e., relatively high concentration of fasteners) and dilute (i.e. relatively low concentration of fasteners, see Section S4, Supporting Information) patterning limits are reported in Figure 4A,B, respectively, using a representative friction coefficient. Interestingly, in the dense system, the order of magnitude of normal forces is double that of the dilute one. Furthermore, the dilute system shows an engagement (and disengagement) kinematics occurring more rapidly than the dense system, assuming a constant engagement (disengagement) speed ∂*y*/∂*t*. This explains why natural fastening solutions are typically based on dilute systems, especially for flying species. Indeed, engagement occurs by inertial forces (proportional to the insect mass) thanks to the sharp deceleration from flying to docking, thus requiring the fastening mechanics to happen in a short time scale or under low compressive loads, as shown by the dilute system.

Moreover, increasing the radius (or decreasing the in‐plane distance between the mating interlockers) and scaling from the dilute to the dense configuration determines an increase in the engagement (and disengagement) forces. Ideally, to increase the effective mechanical work of adhesion and lower the engagement work, *R*/*I*_0_ has to be smaller during approach than during retraction. Therefore, a system showing a dilute configuration (high *I*_0_ values) during approach while being dense (low *I*_0_ values) during retraction will again provide an effective mechanical adhesion with relatively low engagement work. The former approach (controlling the tip radius) has been recently introduced for drug delivery applications,^[^ ^21^ ^]^ the latter (controlling *I*_0_) will be described in Section 2.5.

*Double‐conical interaction*

The rounded geometry in a single mating pair is characterized by a critical in‐plane distance *I*_crit_, determining the transition from an interlocking to a jamming state during approach motion. To avoid jamming, a more effective tip geometry should exhibit a discontinuous profile at its apex, whose angular extension is designed according to the friction value to avoid sticking during the approach motion. The cone is identified as the simplest geometry satisfying this requirement; however, the double‐cone geometry is adopted in order to tailor the contact response both in approach and receding motion (Figure 3A). Furthermore, by varying the angles defining the geometry (α and β), the engagement or disengagement force can be enhanced or decreased for the surface to be adapted to the morphological traits of non‐engineered counter surfaces, such as villi‐patterned intestinal tracts.

**Figure 5:**
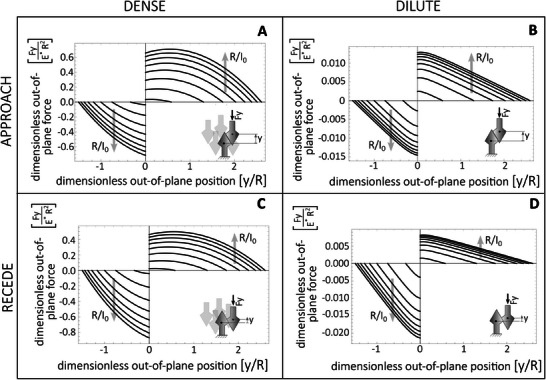
Normalized out‐of‐plane force *F*_y_ during the single‐pair interaction of conical interlocking structures at increasing values of *R*/*I*_0_, spanning from 0.4 to 2, with a spacing of 0.2. A,B, Out‐of‐plane force during approaching motion of densely packed and diluted double‐cones, respectively. C,D, Out‐of‐plane force during receding motion of densely packed and dilute double‐cones, respectively. With cone angles α=π/6 and β=π/3, μ=0.25 (wet environment), *I*_0_=1.5mm, with equivalent Young modulus E*=4MPa.

The model derivation is reported in Section S4.2 (Supporting Information). In **Figure** **5**, the out‐of‐plane force *F*_y_ for the double‐cone tip is illustrated during both approaching (A and B) and receding (C and D) motions, based on a representative friction coefficient. The figure displays the dense and dilute patterning limits on the left and right sides, respectively. The top (bottom) cone angle is π/6 (π/3). Both angles are lower than the static friction angle. As seen in the case of spherical tips, the order of magnitude of the normal forces is higher in the dense system compared to the dilute one. However, no jamming occurs in this context. Furthermore, because the contact angles are different, the single‐pair engagement force is lower than the disengagement one, favoring reversible adhesion with a relatively low engagement force.

#### 2.2.2 Probability Density Function of Mating Microstructures

**Figure 6:**
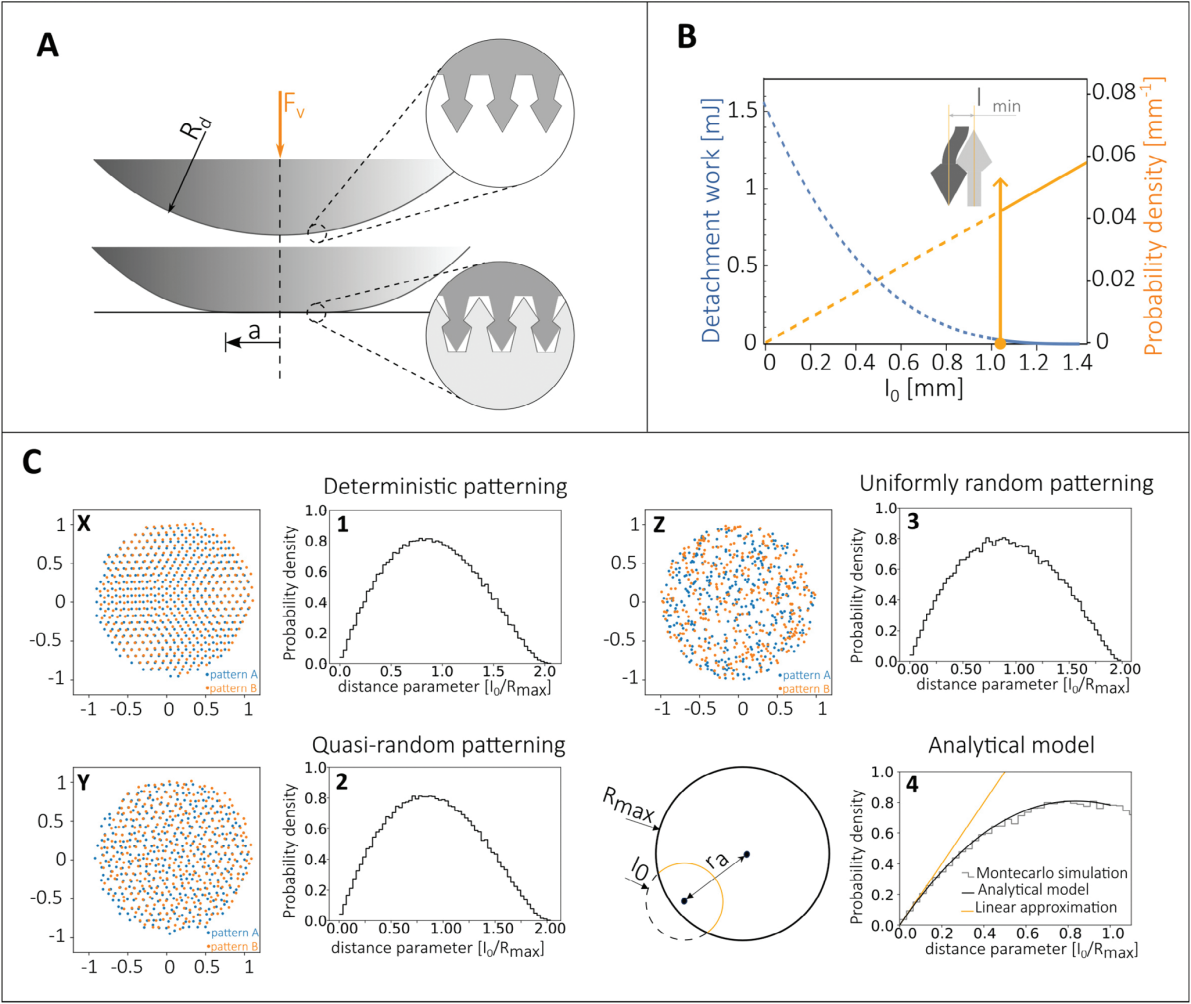
A) Schematics of a generic contact, with magnified microfasteners shown in the insets. In the model, the micro geometries lengthscale is much smaller than the size of the contact, identified here by the semi‐contact length *a*. *F*_v_ is the externally applied squeezing force. B) Work of detachment (blue) and probability density function of the in‐plane distance between two micro geometries after interlocking (orange), as a function of the distance between two micro geometries *I*_0_. The dotted lines represent geometrically non‐physical solutions due to the lack of interlockers in this distance domain because of the finite thickness of their supporting rods. The probability associated with the missing interlockers' population is thus collected by a Dirac's delta located at the threshold *I*_0 min_. C1–C3) Superposition of two patterned unit circular domains (orange and blue dots) with deterministic, quasi‐random, and uniformly‐random patterns (X, Y, Z, respectively) and related histograms (1, 2, 3, respectively) of the in‐plane distances. C4) Geometrical construction of the theory predicting the probability density function of the in‐plane distances between microstructures, and comparison of the theory prediction with the Monte Carlo simulation results.

The probability density function of the distance between mating micro‐structures, defined as *p*(*I*_0_), is required to homogenize the random interactions occurring at the scale of the representative elementary volume of the interface (Figure 2B; Section S5, Supporting Information). *p*(*I*_0_) is thus adopted to calculate the effective work of mechanical adhesion (Section 2.2.3) as well as other emerging behaviors at the macroscale, such as the engagement and disengagement pressures. It is observed that, in the theory formulation, *p*(*I*_0_) takes into account the distribution of distances in the actual (or deformed) contact configuration, as shown in **Figure** **6**B illustrating the double‐conical interlockers example. In the figure, a Dirac's delta identifies the population of micro geometries undergoing in‐plane displacement (up to an in‐plane distance *I*_0min_) upon interlocking. Jamming is also affecting *p*(*I*_0_)'s lower limit in the case of spherical interlockers, as described earlier. Any other interlocker shape is likely to show similar limits to be considered when building the actual distribution of distances.

Different surface patterns are analyzed for a more general application of the theory: A deterministic hexagonal lattice, a uniformly‐random placement distribution, and a quasi‐random placement distribution over a circular area (Figure 6 CX–CZ, respectively). The quasi‐random lattice is obtained by randomly placing points, with Gaussian distribution, around an otherwise deterministic hexagonal lattice. Furthermore, it must be noted that the relative angular alignment of two generic interacting surfaces, patterned with the aforementioned lattices (see Figure 6A), is considered a random variable with uniform distribution. Thus, *I*_0_ corresponds to a random variable also in the case of deterministic patterning. Indeed, assuming a random angular alignment allows the development of a more general theory of fasteners, enabling its applicability to real applications, including biological, i.e. without the constraint of controlling the relative angular positioning of the interacting surfaces. Finally, a finite size system (*R*_MAX_ in Figure 6 C4‐left) is considered in the model to enable predictions for a more generic fastener situation, where the size of the single interlocker might not be significantly smaller than the size of the apparent contact area. Similarly, it is well known in contact mechanics that system finiteness can affect to a large extent the average interface separation as a function of applied contact pressure for a randomly rough contact.^[^ ^74^, ^75^ ^]^

In Section S5 (Supporting Information), *p*(*I*_0_) is derived either from geometrical considerations or with direct Monte Carlo simulations. Representative results are shown for a deterministic hexagonal lattice, a quasi‐random, and a uniformly random positioning distribution in Figure 6 C1–C3, respectively. Figure 6 C4‐right reports the comparison between the analytical model of *p*(*I*_0_) for the uniformly random distribution (solid black line) and the Monte Carlo results (discontinuous histogram), together with the linear approximation of *p*(*I*_0_). Interestingly, despite the different lattice nature, the distribution of distances is qualitatively unaffected, highlighting the key role of the random angular alignment on the generation of *p*(*I*_0_).

#### 2.2.3 Effective Work of Mechanical Adhesion

In Section S6 (Supporting Information), the effective work of mechanical adhesion Δγ_eff_ is determined as a function of the single interlocker work of detachment *W*_int_(*I*_0_) and of the actual distribution of distances *p*(*I*_0_):

(1) $$\Delta \gamma_{\mathrm{eff}}=\pi R_{\max}^{2}\rho_{a}\rho_{b}\int_{0}^{I_{0\max}}dI_{0}\;p\left(I_{0}\right)W_{\mathrm{int}}\left(I_{0}\right)$$

where ρ_a_ and ρ_b_ are the areal densities of interlockers (for the mating surface a and b, respectively, interacting over a circle with radius *R*_max_). For the case of interlockers characterized by a (partial) jamming behavior, such as for spherical tips, the RHS of Equation (1) has to be reduced by the jamming work of the buckled micro fasteners (Section S6, Supporting Information).

The CZM, similarly to Equation (1), can be determined by averaging the out‐of‐plane contact forces *F*_y_, acting among interlocker pairs over the representative elementary volume of the interface. Thus, the average contact pressure σ(*y*) as a function of interlocking position *y* can be obtained, as discussed in Section S6 (Supporting Information), for the approach and removal motion. σ(*y*) is the CZM, or effective interface integrated interaction law, needed to predict the contact mechanics at the application scale, as typically done in the case of randomly rough contact mechanics simulations.^[^ ^76^ ^]^ Furthermore, σ(*y*) is needed to determine the engagement pressure σ_a_, corresponding to the maximum contact pressure under approach motion, as well as the pull‐off pressure σ_d_, the latter corresponding to the interfacial adhesive strength of the patterned surface, which is a key factor in determining the performances of an adhesive patch. Thus, for a ball‐on‐flat contact patterned with random fasteners, mechanical adhesion is activated only for externally applied forces larger than $F_{\mathrm{crit}}=\left(\pi^{3}/6\right)\sigma_{a}^{3}R^{2}/E^{*2}$, where *R* is the ball radius. Below *F*_crit_, a repulsive interaction among mating fasteners occurs all over the macroscopic interaction domain during the whole approaching stage, leading to no detectable adhesion during the removal.

### 2.3 Interlocking Properties of 3D‐printed Bio‐mimicking Architectures

In the following experimental sections, the tests will be conducted with structures in the millimeter scale for ease of fabrication and research sustainability (see Figure 3D,G), allowing us to collect a statistically relevant number of samples for the theoretical validation. However, it is worth noting that the designs investigated in this work (double‐conical and spherical) do not require printing supports, which are difficult to remove when operating at micrometer scales. As a consequence, the structures can be scaled down to a limit that is solely dependent on the resolution allowed by the specific fabrication protocol. Therefore, two demonstration samples have been fabricated employing a two‐photon polymerization technique (Figure 3E,H), showcasing the possibility of covering a broad range of dimensions, from the mm to the microscale.

**Figure 7:**
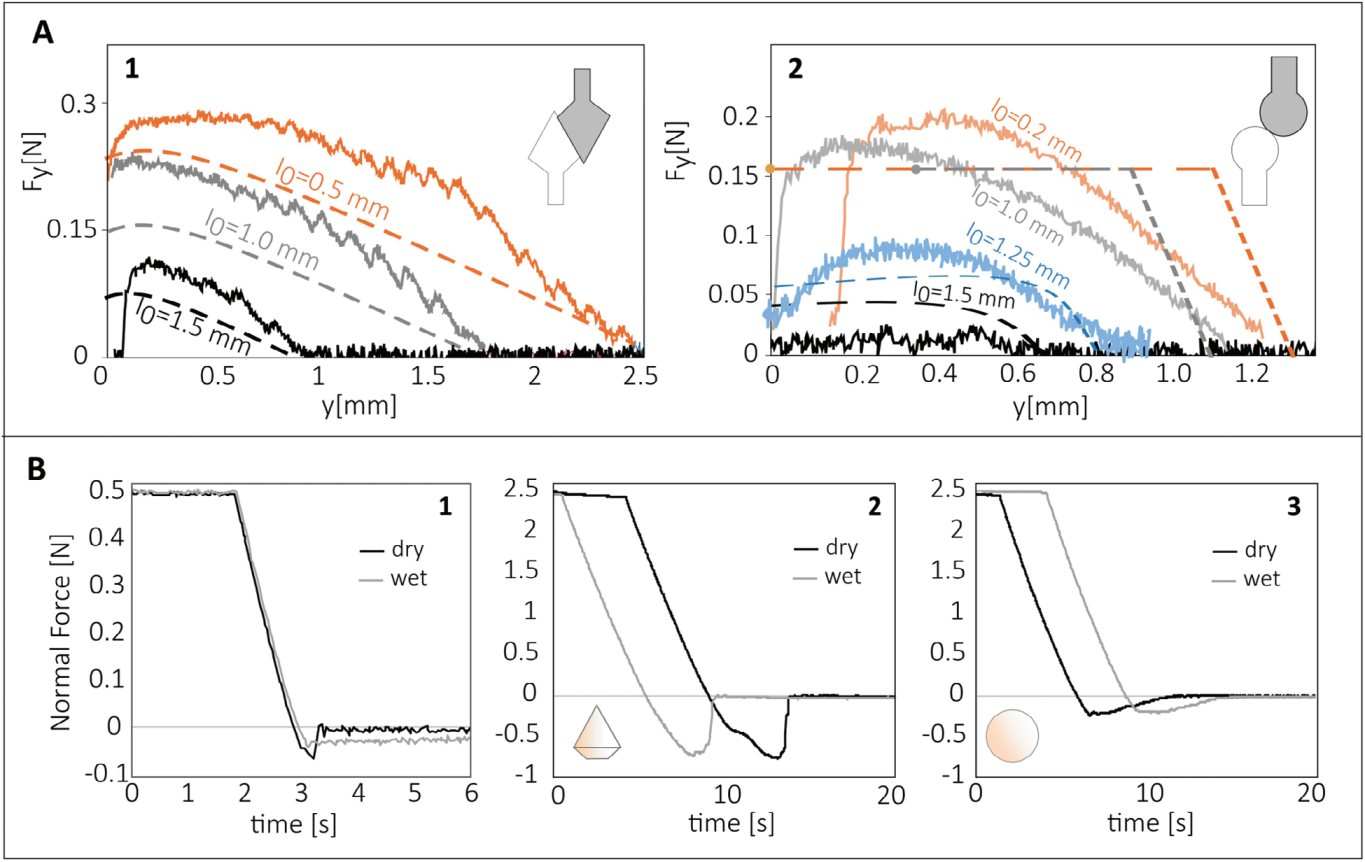
A) Characterization of the single interlocker during approach motion, in terms of contact force vs out‐of‐plane distance, at constant penetration speed of 50 µm s^−1^ and 20°C. A1 and A2, Comparison between experimental (solid lines) and theoretical (dashed lines) results for, respectively, the single conical (*R*=1mm, α=π/6, β=π/3) and spherical (*R*=1mm) interlockers, at various lattice distances; the contact pair materials are polymer‐A and polymer‐B (polymer‐A and polymer‐B are defined in the Materials Section in Experimental Section). B) Contact force vs displacement in a typical pull‐off experiment in dry (black line) and wet (grey line, with distilled water) conditions, at a constant retraction speed of 50 µm s^−1^ and 20° C. In particular, B1, JKR adhesion test between a smooth, soft sphere (radius 36.8 mm) 3D printed with polymer‐A and a smooth flat sheet 3D printed with polymer‐B. B2 similar to B1 but for a polymer‐B sphere (*R*_d_=40mm) in contact with a polymer‐A sheet, both patterned with a population of double‐cone structures (*R*=1mm, α=π/6, β=π/3). B3 similar to B2 but for a polymer‐B sphere (*R*_d_=40mm) in contact with a polymer‐A sheet, both patterned with a population of spherical structures (*R*=1mm). Both B2 and B3 have a patterning areal density of 0.18 mm^−2^.

The experimental contact mechanics at the scale of the single‐pair interaction, together with their collective contact behavior resulting from a macroscopic ball‐on‐flat contact, are reported in **Figure** **7**. In particular, the contact force vs. out‐of‐plane distance (solid lines) between the micro‐geometries of a single contact pair is reported in Figure 7A for both conical (A1) and spherical (A2) tips, at different in‐plane distances *I*_0_, during approach. The experimental results for both micro‐geometries are compared with theoretical predictions (shown as dashed lines), which are obtained by using measured friction values on larger printed samples, as detailed in Section S1 (Supporting Information). This comparison confirms the validity of the model's main assumptions and its ability to represent small‐scale contact mechanics. Deviations from the experiments are likely due to neglecting the contact‐induced rotation of the microstructures, and to the non‐linear response of the supporting rods when subjected to large deformations.

**Figure 8:**
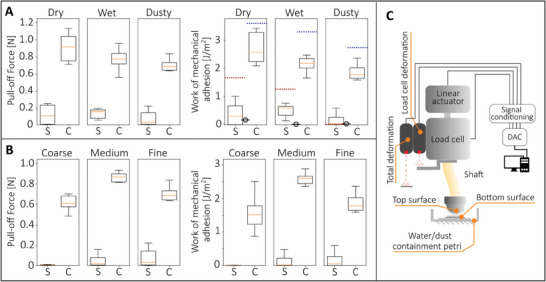
In all the experiments, the contact occurs between a patterned sphere (with radius 40*mm*, polymer‐B) and a flat, patterned sample (polymer‐A). The patterning is made by spherical (indicated by S in the figure) and double‐conical (indicated by C) tips, with the same areal density of 0.18 mm^−2^, and they all have a radius of 0.7mm. The boxes on the box‐plot graph extend from the first quartile to the third quartile of the data. The whiskers extend from the box to the farthest data point, lying within 1.5 times the inter‐quartile range from the box. The (experimental) work of mechanical adhesion has been extracted as the integral of the out‐of‐plane force during the detachment process divided by the area of the sample; indeed, in the tested samples the whole patterned area contributed to interlocking, i.e. the penetration of the microstructures occurred in every region of the sample. A‐left, box‐plots (*n*=10) of experimental results of the pull‐off force and A‐right, work of mechanical adhesion of patterned surfaces in dry, wet, and dusty conditions. The dotted horizontal lines represent the theoretical predictions (red for spherical structures and blue for conical structures). The signed circle marks represent the work of adhesion measured for a smooth 40 mm diameter polymer‐A sphere in contact with a smooth polymer‐B flat sheet. B‐left, experimental pull‐off force, and B‐right, effective work of mechanical adhesion, for different dust size distributions (from coarse to fine, see Figure S2, Supporting Information). Box‐plots (*n*=10) for spherical (S) and double‐conical (C) tips, upon ten measurement replications. C, Schematics of the set‐up used for the tests, with the indication of the main actuation and sensing devices. The surfaces are brought in contact by a linear actuator that moves the load cell and the position sensors, the real relative displacement used to calculate the detachment work is the difference between the laser sensors readings as explained in more detail in the Experimental Section.

The case of spherical tips is shown in Figure 7 A2. When the spherical structures are pressed together, the supporting rod deforms axially until the critical buckling load is reached. Afterward, the structure continues deforming by bending, keeping the critical (buckling) load constant. This happens only when *I*_0_, the inter‐axis distance between mating structures, is sufficiently small, say less than a critical distance *I*_crit_ (Section S4.1.3, Supporting Information). *I*_crit_ is very sensitive to the friction occurring among the printed geometries: Their residual roughness can affect such a threshold as well as the same jamming dynamics. The latter has been approximately included in the theory (Section S4.1.3, Supporting Information), which is still able to capture the order of magnitude of the axial forces without the adoption of fitting parameters. Furthermore, the residual roughness also determines the discontinuous, saw‐tooth‐like behavior shown by the experimental results in Figure 7 A1. In this case, the adopted 3D‐printing fabrication process adds a geometrical layering to the fasteners, which affects their interlocking dynamics. The residual topography in the 3D printed interlockers seen in Figure SS3 (Supporting Information) is therefore responsible for increasing the effective friction coefficient during the single pair interlocking. For this reason, it is hypothesized that jamming occurs more frequently for both spheres and double conical structures than predicted by the theory presented in Section S4.1.3 (Supporting Information), thus reducing the number of interlocked structures. This can explain why for single interaction tests, the experimental *F*_y_ is higher than the theoretical one (see Figure 7 A1 and A2) as opposed to the results in **Figure** **8**, where theory predicts a higher work of mechanical adhesion.

In Figure 7B, the contact force as a function of the removal time is reported for a ball‐on‐flat pull‐off experiment. In particular, in B1, both the sphere and the flat counter sample are unpatterned (say smooth), whereas in B2 and B3, both the surfaces are patterned with micro‐cones or ‐spheres, respectively. The nominal ball radius and the materials are the same in all the experiments; the black (grey) line indicates a dry (wet, microstructures completely submerged in lubricant) interaction. More details about the surfaces are reported in the figure caption. Interestingly, the pull‐off force, thus the effective work of adhesion, is negligible for the wet contact, where the negative (attractive) force during removal is only due to the capillary contribution to adhesion, not existing in fully underwater applications. On the other side, the adoption of micro‐geometries overcomes this limitation, leading to pull‐off forces insensitive to the local adhesion (Figure 7 B2,B3). This fully agrees with the theoretical findings, see Figure S4 (Supporting Information), i.e. adhesion does not quantitatively affect the contact mechanics of a single interlocking pair and the overall mechanical adhesion. Furthermore, despite the reduced (but not suppressed) sliding friction from the dry to the wet contact case (see Figure S1A,B, Supporting Information), the pull‐off forces are only slightly affected, Figure 7 B2,B3, confirming the leading role of the micro geometry contact mechanics on the overall mechanical adhesion.

Of the observed patterns, jamming primarily affects the spherical fasteners (Figure 7 B3), resulting in a reduced pull‐off force compared to the conical tips (Figure 7 B2). This happens despite spherical tips exhibiting a larger disengagement force than conical tips (Figures 4 and 5). Jamming, indeed, introduces a cut‐off in the probability density function of interlockers distance, thus limiting the true areal density of engaging tips. Finally, it is observed that discrepancies between the theory and experimental findings can also be ascribed to differences between the friction coefficient adopted in the calculation (Figure S1A, Supporting Information) and the friction locally occurring in the engaging fasteners, which is sensitive to the specific geometry being printed.

### 2.4 Bioinspiration for Adhesion in Underwater and Dusty Environments

Dusty and underwater environments pose significant challenges for adhesion due to the presence of dust particles or water layers, which hinder adhesion. The performances of both spherical and conical interlockers have been thus experimentally evaluated for the case of dry, wet, and dusty contact environments (Figure 8A,B), thanks to the development of an ad hoc testing setup, see Figure 8C and Experimental Section. In particular, both the pull‐off force and the work of mechanical adhesion are shown for the spherical (S) and double‐conical tips (C) in Figure 8A. As expected, the spherical fasteners exhibit lower interlocking performance compared to the double‐cones, independent of the contact environment. The latter quantitatively affects the mechanical adhesion for the double‐cone tip, with the dusty case showing the lowest value. In contrast, the spherical tip is less sensitive to the environment. The true work of adhesion (measured for the smooth contact) is also shown in the Figure with the signed circle mark. For the adopted (unoptimized) spherical geometries, the achieved effective adhesion is of the same order of magnitude as the true work of adhesion, as expected from Figure 7.

The dusty case is further investigated by fabricating dust with different probability distributions of particle size, whose characterization is reported in Section S2 (Supporting Information). In particular, dust with coarse, medium, and fine size is distributed at the contact interface, and the pull‐off experiment is executed for both the spherical and double‐cone interlockers, see Figure 8B. For the spherical tip, the coarse dust particle suppresses the work of mechanical adhesion due to the further jamming induced upon contact between interlockers and dust particles. On the other hand, double‐cone interlockers are mostly insensitive to the dust size distribution due to the lack of jamming effect. The cone geometry effectively accommodates larger particles by encapsulating them within the lattice structure, minimizing their impact on overall performance, regardless of the dust grain size dimension (Figure 8B; Figure SS2, Supporting Information for the dust size characterization). In particular, the encapsulation of larger grains does not affect adhesion up to a certain threshold grain size. The latter mostly depends on the residual gap between mating structures in the interlocked configuration. In particular, a residual volume is left among interlockers, which can host an equal volume of contaminants. Thus, particle size or particle volume higher than this threshold will be detrimental for adhesion, due to the third body interaction with the grains. However, since this obstruction phenomenon is entirely dependent on geometrical descriptors, the dimensions of the interlockers can be designed to accommodate a specific dust particle distribution.

The contact phenomena occurring at the REV scale can thus turn around the contact performance detectable at the scale of the single scale interaction; in particular, the pull‐off force at the single spherical pair contact is much larger than the corresponding values for the double‐cone interaction (see Figures 4 and 5). However, the contact statistics at REV scale are strongly affected by the probability distribution of interlocker distances and their related jamming behavior, resulting in an opposite performance at the application scale. These findings underscore the limitations of the single‐pair contact model, which, alone, cannot predict the fastening forces at the application scale, requiring the development of multiscale contact mechanics. Predictions from our multiscale approach are shown as dashed lines in Figure 8A‐right, exhibiting qualitative agreement with the experimental results. Dust, in particular, strongly reduces friction (see Section S1, Supporting Information). Both the model and experimental results demonstrate that interlocking structures offer a promising strategy for achieving robust adhesion in these adverse conditions, allowing the development of tunable adhesive devices.

### 2.5 Bioinspiration for Micro‐grasping and ‐sorting

Biological systems show that substrate‐selective attachment is key to avoiding the danger of cross‐species reproduction.^[^ ^77^, ^78^ ^]^ It is also useful when detaching a section of the body is required to flee from a predator.^[^ ^15^ ^]^ Similar to the key‐lock mechanism, this biological principle can be technologically exploited to selectively adhere a patterned sample to target objects. By employing an optimized design of the microstructures, mechanical adhesion can be activated against objects with specific geometrical or mechanical characteristics, or the ability to switch and control adhesion can be enabled.

**Figure 9:**
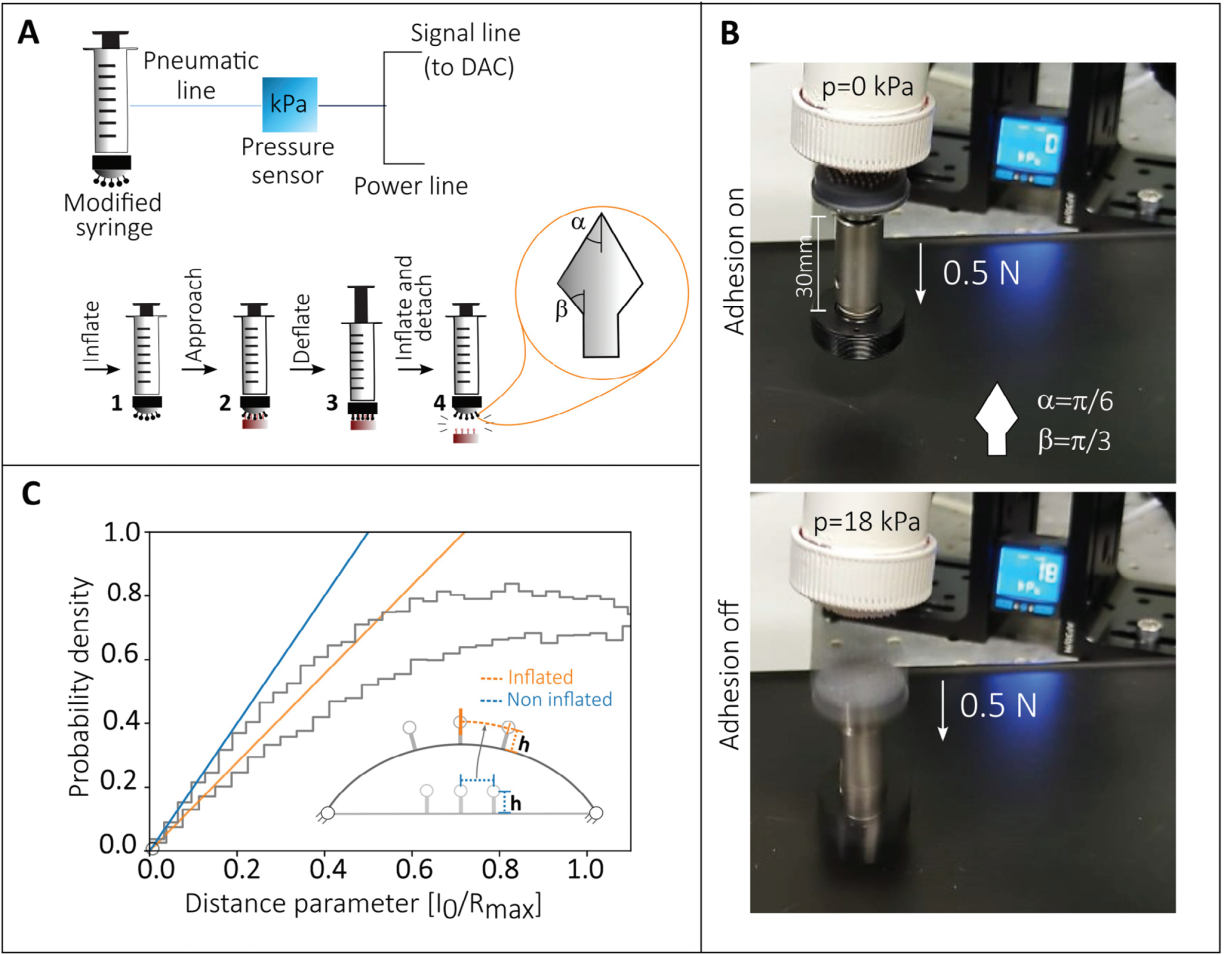
A) Schematics of the adhesion‐switching prototype setup (top), and procedure to attach/detach samples using substrate inflation (bottom): 1) The modified syringe inflates the patterned membrane. 2) Approach and interlock with the target sample. 3) Deflation of the membrane to secure the fastening. 4) Inflation to release the target sample when needed. B, Pressure reading (background digital blue screen) during adhesion switching for the lifting/dropping of a 0.5N weight. C) Effect of substrate inflation on the probability density function of distances between structures, *p*(*I*_0_). Upon inflation with a pressure of 18kPa, the distance among micro geometries increases, leading to the release of the interlockers.

In **Figure** **9**A, the schematics of the experimental procedure adopted to provide switchable adhesion in a real application are reported. In particular, Figure 9A shows a setup composed of a syringe modified to inflate a soft, polymer‐A patterned membrane and a pressure gauge to measure the inflation pressure. Inflating the soft membrane determines the variation in the distance distribution between mating fasteners *p*(*I*_0_) (Figure S5.3C and Section S5.3, Supporting Information) thus intimately affects their work of mechanical adhesion, as well as the engagement pressure. In Figure S5.3A‐bottom (Supporting Information), the patterned membrane is inflated 1) through the syringe, leading to the new distance distribution of mating structures *p*(*I*_0_) as shown in Figure S5.3C (Supporting Information). The latter results in a relatively low engagement force, allowing interlocking 2) with a reduced work of attachment. The successive deflation restores the distance distribution to the original curve to secure the fastening to pull‐off force designed for the specific application 3). Eventually, the fastening can be reversibly released (switched off) by inflating the membrane 4). With this approach, a small demonstrator with an apparent interaction area of 2.5 cm^2^ was built and adopted to alternately lift and release a 0.5 N weight, as shown in Figure S5.3B (Supporting Information).

**Figure 10:**
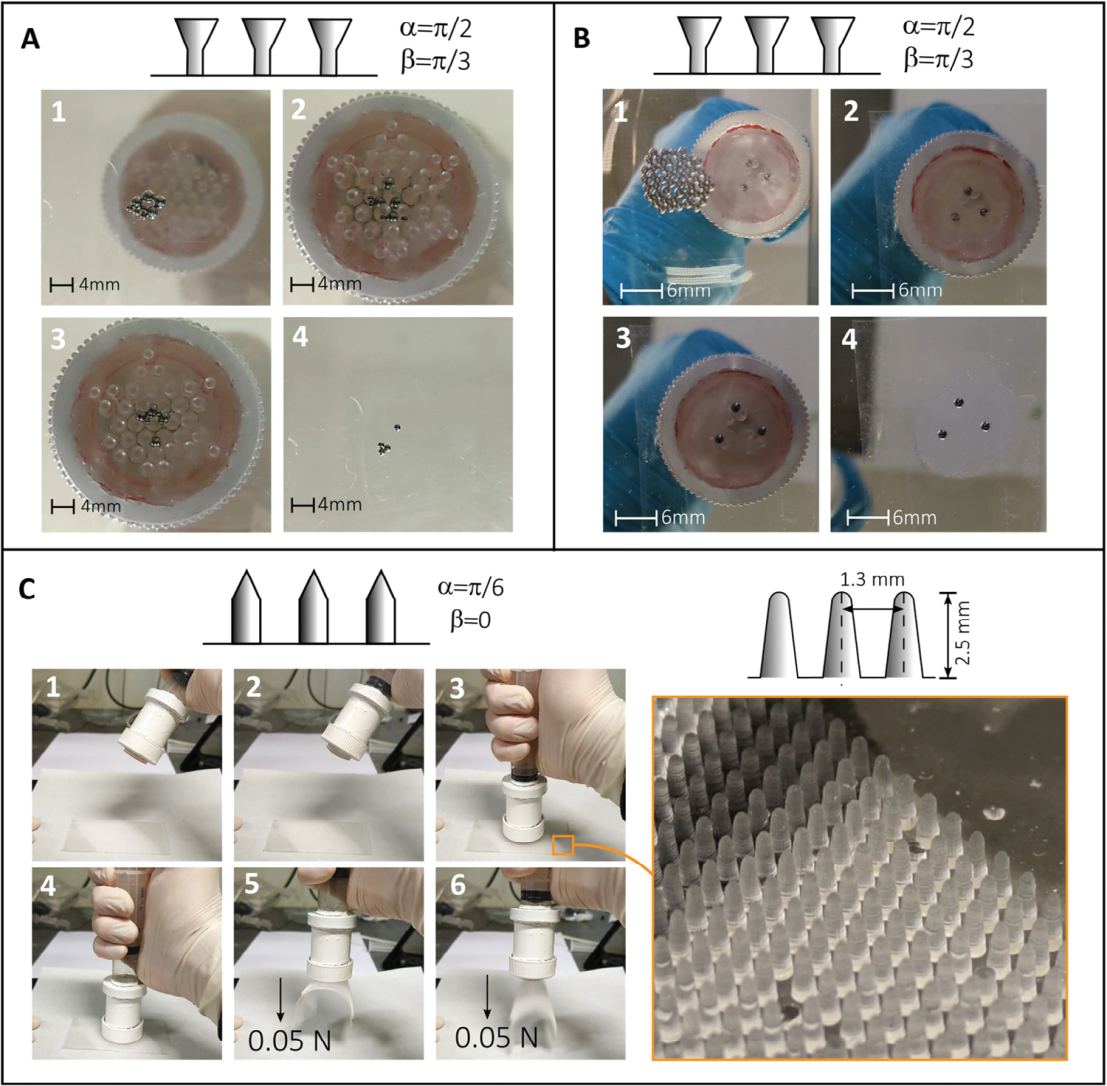
A) Selective picking on 8 different diameter steel balls (4 × 1mm, 4 × 2mm) using a specifically designed conical interlocker. B) Pattern grasping on 1.5 mm steel balls. The membrane is equipped with a triangle‐shaped pattern that can grab steel balls following the triangular shape; they are then released on a substrate covered with double‐sided tape to visualize the release points. C) Switching adhesion procedure on 1:20 PDMS villi‐shaped structures through the fabrication of a specifically designed conical interlocker.

The aforementioned active *p*(*I*_0_) approach can also be adopted to achieve accurate and selective grasping, lifting, and releasing of multiple specific targets, as shown in **Figure** **10**A. In particular, the setup described in Figure S5.3A (Supporting Information) was equipped with a particularization of a double‐conical microstructured membrane (Figure 10A‐top). The lattice distance and the microfasteners' radii were chosen to allow the deflated membrane to interlock with 2 mm diameter bearings steel balls but not with 1 mm ones. Initially, all the spheres are mixed up on a transparent plate, whereas the membrane is inflated without reaching contact (Figure 10A‐1). After the approaching phase, the spheres interlock with the microstructures on the still‐inflated membrane independently of their radii (2); the successive deflation changes the distance distribution of the microstructures, leading to the selective grasping of the larger balls (3). Finally, upon tool removal, only the 1 mm spheres are left on the transparent substrate (4). In Figure 10B, a similar approach is used to easily grasp a set of three steel spheres and release them accordingly to a predefined pattern (triangular in the demo, for simplicity): After inflation and contact of the patterned membrane with a pool of spheres, the syringe is deflated and the spheres grasped from the pool (1) and transferred to a second substrate covered with a double‐sided tape (2). The syringe is finally inflated, letting the spheres fall on the tape (3). The tape is needed only to prevent the steel spheres from rolling off the bottom substrate after their release from the grasping surface, preserving the designed arrangement (4).

In this context, inflation pressure is adopted to trigger or induce a controlled variation of the interlockers' areal density. This approach can be advantageous over other strategies that exploit slow and difficult‐to‐control diffusive processes to trigger the adhesion mechanism.^[^ ^26^, ^28^ ^]^ However, any other external trigger stimulus would be effective to induce an areal density variation, e.g., by fabricating interlockers on the top of an electroactive (stretchable) polymer sheet or a piezoelectric sheet.

### 2.6 Bioinspiration for Tissue Adhesion

Inspired by the geometry of intestinal villi,^[^ ^79^ ^]^ the scaled‐up version (see Figure 10C‐right) of these protrusions was fabricated using soft lithography to explore the potential of interlocking patches for tissue attachment. The typical geometry of a villus‐like structure prevents local (normal) contact forces from contributing to mechanical adhesion, thus letting friction forces withstand the entire interlocking load. To optimize interlocking while preventing sticking and subsequent jamming, a sharp corner at the tip of microstructures was introduced (see Figure 10C‐left, top), resulting in another particularization of the versatile double conical shape. In this case, the bottom cone β has an opening angle of 0 rad. Although the interlocker geometry is less effective for interlocking than the previously discussed optimized double‐cone of Figure S5.3B (Supporting Information), it provides enough lifting capacity to hold the target samples (0.05N weight). Villi‐like adherence is obtained as follows: In Figure 10C, 1) the cone‐patterned membrane is initially deflated; 2) acting on the piston to inflate the membrane allows the interlockers distribution to change accordingly; 3) the membrane is then brought in contact and pressed onto the target allowing the interlocking to occur; 4) the syringe piston is then lifted again resulting in membrane deflation; 5) allowing the fastening and lifting of the villi‐like sample; 6)which can be eventually released upon subsequent inflation. As expected from the theory, this experiment shows that interlocking performance is reduced when both interacting surfaces exhibit a relatively low elastic modulus. In this demo, indeed, the surfaces designed to emulate the structure of intestinal villi were fabricated from a soft PDMS mixture (1:20 weight ratio) to mimic the elasticity of human internal tissue (Young's modulus ≈ 1MPa), which reduces the overall lifting capacity compared to the optimized double‐conical shape in Figure S5.3B (Supporting Information).

## 3 Conclusion

Mechanical interlocking is an effective approach to deliver tunable and controllable adhesion in harsh environments. Nevertheless, effective adhesion can be obtained only upon a proper engineering of both the microscale contact geometry, constituted by the single‐pair contact, and their collective interaction occurring toward the lengthscale of the application. Thus, a comprehensive multiscale theory has been introduced to link the contact mechanics at the single interaction scale to the macroscale of the application by developing a statistical description of the interactions occurring at the representative elementary volume of interfaces.

Two fastener geometries have been analyzed, the spherical and the double‐cone tip. A range of microscale phenomena, such as contact sticking and interlockers buckling, as well as collective phenomena, such as jamming, have been investigated, the latter causing a reduced mechanical adhesion. The fastening statistics and the probability distribution of distances among interlockers were also analytically derived and successfully compared to numerical results.

Theoretical predictions were validated against experimental results obtained from an in‐house built setup, allowing for accurate measurement of the contact force and the detachment work in dry, underwater, and dusty environments. Interestingly, it is shown that the arrangement of interlocking structures, either hexagonal, quasi‐random, or random, does not substantially affect the probability distribution of interlockers' distance if the fastening among surfaces occurs with random in‐contact plane orientation.

Furthermore, switchable mechanical adhesion, induced by a controllable variation of the probability density function of interlockers distance, is shown to be effective in micro‐grasping, ‐sorting, and tissue‐specific adhesion. The design and fabrication approaches developed in this work can pave the way for the effective and reliable application of mechanical adhesion technology in several fields, from the food and electronics industry (pick&place mechanisms, selective picking, reliable joint locking) to bio‐medical applications (adhesive, tissue‐specific patches for monitoring and drug delivery), space and harsh environment applications, such as underwater or dusty applications, where chemical adhesion is suppressed. This work lays the foundations for a comprehensive design of interlocking surfaces, identifying the factors that play a key role in mechanical adhesion.

## 4 Experimental Section

### Materials

Patterned samples have been produced with Formlabs resins: Elastic 50A v1 (polymer‐A in the manuscript, elastic modulus *E* ∼ 3 MPa), Tough 2000 v1 (polymer‐B elastic modulus *E* ∼ 1.2 GPa), and High Temp v2. For the surface covered with villi‐like geometries, the Sylgard 184 elastomer kit (Dow Corning) was used, mixing curing agent and pre‐polymer with a weight ratio of 1:10 for the mold, and 1:20 for the patterned surface.

Wet adhesion tests were carried out using distilled water after rinsing the samples with copious amounts of distilled water and isopropyl alcohol. To recreate dusty environments, two dust particles were used, i.e. talc powder for fine grains and clay powder at various grain sizes for the coarse grains, see Figure S2 (Supporting Information), obtained using a 100 µ*m* sieve and mechanical shaking. The steel spheres used for selective picking are standard bearing balls with a diameter of 1 and 2 mm.

### Fabrication

Adhesion test samples have been fabricated through 3D printing with a Formlabs 3B+, a laser SLA 3D printer, using a relatively stiff polymer‐B for the top patterned sample, and a soft polymer‐A for the bottom patterned sample. The soft rod connecting the load cell to the top sample was printed using the polymer‐A resin, thus giving additional rotational degrees of freedom to the sample during the engaging stage (see Figure 8C). During wet tests, the patterned surfaces were fully submerged in distilled water, whereas for testing in dusty environments, the dust poured in the contact was weighed with a precision scale (35 mg) before each test.

For the fabrication of the villi‐like surface, a molding technique has been used: A first positive replica has been printed with a high precision resin (High temp v2 from Formlabs) for high temperatures, then a 2nd (negative) mold made of Sylgard 184 PDMS with mixing ration 1:10 was generated from the former. Finally, Sylgard 184 PDMS with a mixing ratio of 1:20 was used again with the negative mold to fabricate the final villi‐patterned surface. On each mold, a parylene‐C film deposition of ≈1.5µ*m* was used to let the obtained surfaces detach.

### Characterization

Adhesion tests have been carried out through an in‐house built tribometer made up of a vertical (z‐axis) linear stage, a load cell to characterize the mechanical adhesion and related contact forces, a horizontal (x‐axis, y‐axis) stage for the positioning of the samples, and a pair of position sensors to read the z displacement of the top sample. The position sensor readings (Figure 8C) are denoted by *p*_1_ and *p*_2_. They have been used to calculate the real experimental penetration value (*y*) of the samples as *y* = *p*_1_ − *p*_2_. This procedure was necessary to exclude the contribution of the load cell deformation. The rod connecting the samples to the load cell was made of a soft polymer (Polymer‐A) to guarantee additional degrees of freedom to the sample, which in this way could interlock in the most favorable position.

Friction characterization tests in Figure S1 (Supporting Information) were done using an in‐house built tribometer, constituted by the same microscope horizontal (x‐axis, y‐axis) stage used for adhesion tests for the positioning and sliding of the samples. It is equipped with an aluminum sample holder, which keeps the sample in contact with the other testing substrate, applying a controlled constant normal force. When the x‐y stage slides, the tangential force deforms the sample holder, and a capacitive position sensor reads the deformation value, which, after proper calibration, can be linearly related to the tangential force acting at the contact.

For the switchable adhesion demonstrator (see Figure S5.3A, Supporting Information), a syringe has been used. It has been modified with a pressure sensor mounted through the piston and a 3D printed adapter to attach a threaded hollow cap, in which the inflating interlocking soft surfaces can be inserted. The setup is connected to a data acquisition card to acquire the signal and a 12 V power supply. The particle‐size analysis has been carried out through an inverted microscope and an in‐house developed software for image analysis (see Figure SS2, Supporting Information).

### Calculations

Monte Carlo simulations were performed using Python scripts; the resolution of the theoretical model for the single interaction was obtained through Wolfram Mathematica scripts. They are available through the repository in ref. [80].

### Statistical Analysis

The experimental data presented in Figure 8 have been cleaned from outliers (points outside the region delimited by the farthest data points within 1.5 times the interquartile range), they have been represented in a box‐plot form, with the orange centreline indicating the median of the sample, and the box height equal to the interquartile range of the sample. Ten observations were collected for each sample, and the resulting box plots were generated using custom Python scripts.

## Conflict of Interest

The authors declare no conflict of interest.

## Author Contributions

M.B. and M.S. contributed to the conceptualization of the study. The methodology and investigation were carried out by M.B., M.S., and L.P. M.B. was responsible for visualization, while supervision was provided by M.S., S.G., and M.D.V. The original draft was written by M.B., M.S., and S.G., and the review and editing were done by M.B., M.S., L.P., S.G., and M.D.V. All authors discussed the results and approved the final version of the manuscript.

## Supporting information

Supporting Information
