# Supplementary material for: Bio‐Inspired Interlocking Micro‐Patterning for Tunable, Switchable and Selective Adhesion in Wet and Dusty Environments
Source: Small. 2025 Feb 26;21(24):2410527. doi: 10.1002/smll.202410527 (PMC12177846; doi:10.1002/smll.202410527)
Supplement: Supplementary file 1 — Supporting Information [file SMLL-21-2410527-s001.pdf]

NANO · MICRO
small

Supporting Information

for *Small*, DOI 10.1002/smll.202410527

Bio-Inspired Interlocking Micro-Patterning for Tunable, Switchable and Selective Adhesion in Wet and Dusty Environments

Marco Bruno, Luigi Portaluri, Massimo De Vittorio, Stanislav Gorb and Michele Scaraggi**

Supplementary Materials for

Bio-inspired interlocking micro-patterning for tunable, switchable and selective adhesion in wet and dusty environments

Marco Bruno, Luigi Portaluri, Massimo De Vittorio, Stanislav Gorb, Michele Scaraggi**

Corresponding authors: marco.bruno@iit.it, michele.scaraggi@unisalento.it

Symbols

α	Tip cone opening angle
β	Bottom cone opening angle
γ_{eff}	Effective work of adhesion
δ	Penetration during contact
$\Delta\gamma$	Surface energy
μ	Friction coefficient
$\sigma(y)$	Averaged normal stress on the control area
a	Contact width
E^*	Equivalent Young modulus
E_{rod}	Young modulus of the supporting rod
F_{el}	In-plane force (e.g. elastic force due to nearby contacting structures)
F_n	Force normal to contact plane
f_n	Normal force per unit length in cylindrical contact
F_t	Force tangent to contact plane
F_y	Out-of-plane force
I	Interaxis distance in the deformed state between mating structures
I_0	Interaxis distance in the undeformed state between mating structures
I_{crit}	Critical interaxis distance that induces jamming-induced buckling
J_{rod}	Second moment of area of the rod cross section
k	bending stiffness of the supporting rod
l	Contact extension in conical contact
l_{rod}	Length of the supporting rod
n_a	Density of structures per unit area on the surface a
n_b	Density of structures per unit area on the surface b
$p(I_0)$	Probability density function of distance between structures
$P_a(x)$	Probability density function of the position of a structure belonging to surface a in the control area
$P_b(x)$	Probability density function of the position of a structure belonging to surface b in the control area
s	Plate thickness
w	Out-of-plane displacement of a plate after inflation
W_{sing}	Work done by the out-of-plane force during the interaction between structures
x	Position vector in the control area
y	Out-of-plane distance between mating structures

1 Friction of unpatterned surfaces

To characterise the friction coefficient available in the contact pairs, a ball-on-flat sliding contact geometry is adopted, Figure S1. In particular, the ball geometry is made by a PDMS hemisphere (R=19mm) coated with a thin sheet of polymer-A, whereas the flat substrate is made by polymer-B film. Both polymer films have been printed such that their orientation during the printing process produces a residual topography approximately similar to the one on the specimens used during interlocking tests.
The characterisation tests have been carried out on a fully automated in-house built setup in dry, wet and dust contaminated conditions; the setup is capable of acquiring multiple variables such as friction force, sliding distance, images of the contact area, and other tribological parameters. A short description of the setup is summarised in the Section Materials and Methods of the main manuscript.

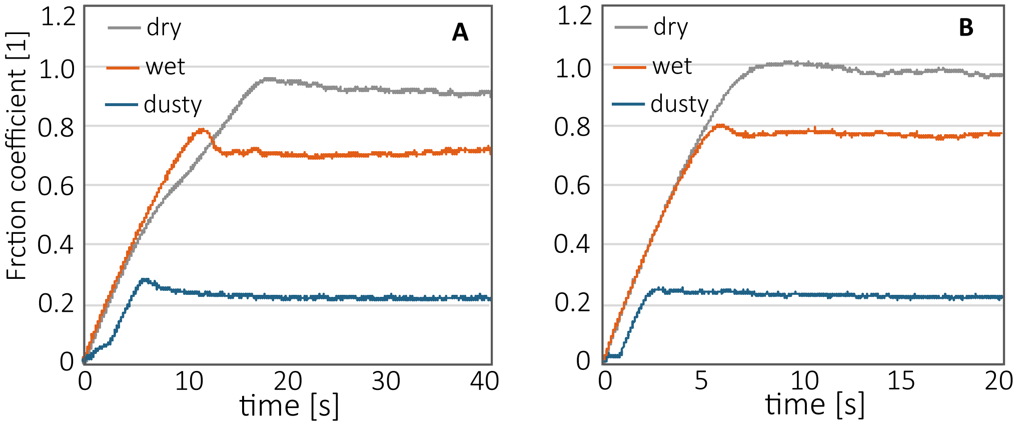

Figure S1: *Experimental results.* A: Friction coefficient in a ball-on-flat contact, of polymer-A (Elastic 50A) in steady sliding contact over a flat polymer-B (Tough 2000) sample, with 1N normal load, sliding speed 0.05 mm/s (A) and 0.1 mm/s (B). In dry, wet and dusty conditions.

2 Fabrication of dusty environments

For dust contamination tests, two kinds of dust have been used: Talc powder (fine size), and clay (medium and coarse sizes). Different sizes have been obtained by manually grinding clay and using a sieve to filter the grains. Dust size have been determined and the estimated volume fraction distribution has been subsequently extrapolated through image analysis with in-house developed python scripts, see Figure S2. In the figure, dust size is characterised before grinding (A) and after grinding (B). Talc powder size distribution in reported in Figure S2C. Optical acquisitions of the dust were taken at 2X magnification and were subsequently analysed, with an image analysis python script, to isolate the particle boundaries and to calculate the equivalent diameter for any closed contour. The plotted distributions represent the estimated volume fraction associated to the equivalent diameter: The estimated volume fraction of the ith diameter range is calculated as the sum of the equivalent volumes in that i^{th} range over the sum of all the equivalent volumes.

3 Residual topography of micro-patterned surfaces

The residual topography on the test samples is related to the fabrication techniques. Every sample used in the tests and demonstrations has been 3D printed, or comes from a replica of a 3D printed specimen. The result is a pattern at the microscopic level that has a big influence on friction and adhesion tests. Differences (in friction and adhesion) could be higher when the geometries of the 3D printed samples are

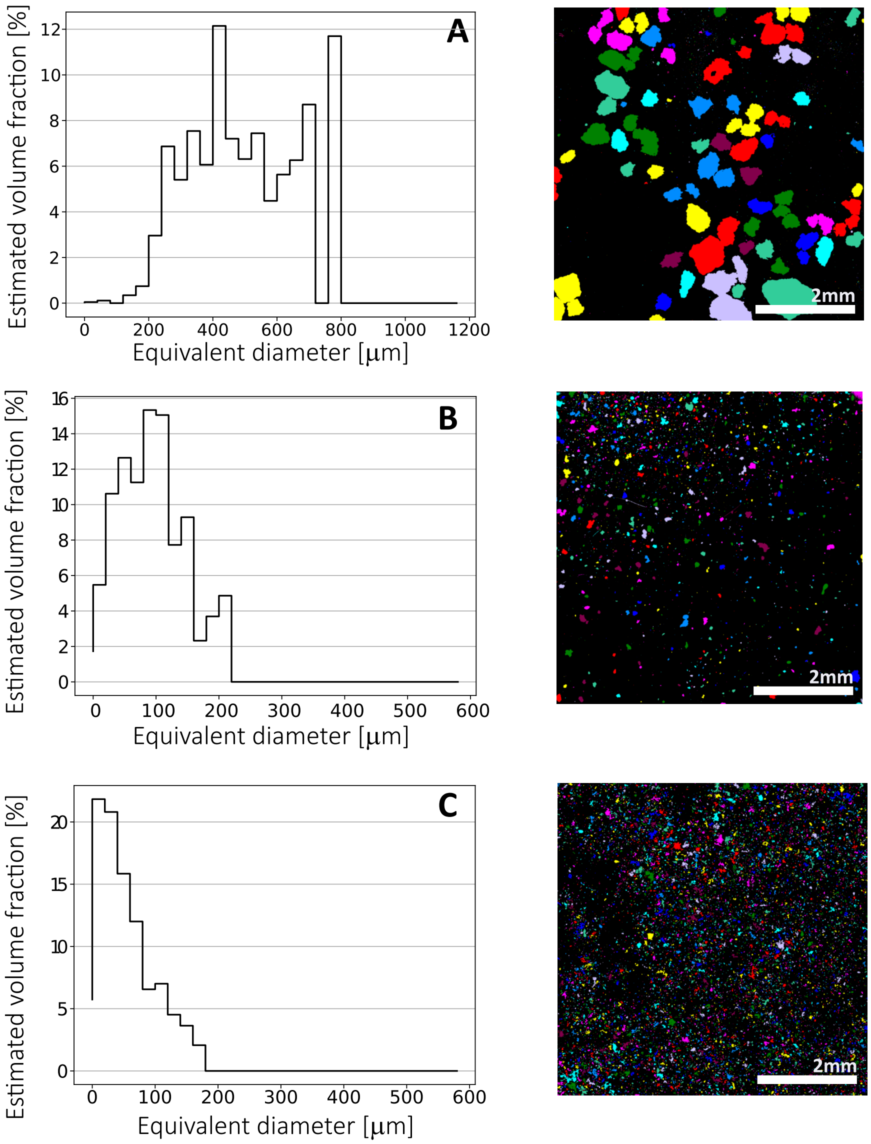

Figure S2: *Experimental results.* Dust-size characterisation through image analysis of clay dust before grinding (A) and after grinding (B), and talc powder (C). The optical acquisitions were taken at 2X magnification and were subsequently analysed through a python script isolating the boundaries and calculating an equivalent diameter for any closed contour. The plotted distributions represent the estimated volume fraction associated to that equivalent diameter.

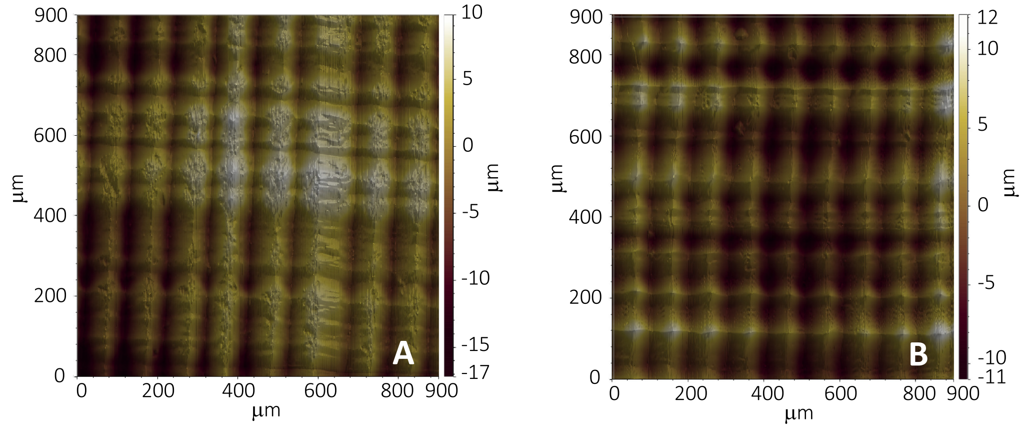

Figure S3: *Experimental results.* A, Residual topography of the 3D printed tough material used to produce the interlocking samples. B, Residual topography of the 3D printed soft material used to produce the interlocking samples. These measurements were taken through a stylus profilometer with a tip radius 2 μm and applied load 1.5mg. The sampling length is $1\mu m$ both for x and y directions.

complex, since the residual topography can be slightly different depending on the angle of the printed surface with respect to the printing direction. Since the measured friction coefficient can vary depending on the geometrical features of the printed object, there will be a difference between the characterization tests and the validation tests on interlockers.

4 Contact mechanics theory for the interlocking single-pair

The generic interlocking single-pair is shown in the schematic of Figure 3, with indication of the micro-geometries adopted in this study, i.e. the spherical and conical tip (Figure 3A and 3B, respectively). Their supporting rod deformation modes are schematically represented in Figure 3C. The local contact, assumed quasi-static, is based on the Hertzian theory [1, 2]. Frictional dissipation is modelled with the classical Da Vinci-Coulomb law, however, any other description of the frictional behaviour could be equally adopted. Furthermore, the local (say van der Waals) adhesion is taken into account. F_{n} and F_{t} correspond, respectively, to the normal and tangential local contact forces; δ, a and l are the contact penetration, the contact radius for spherical contact, and the representative length of the contact area for conical contact, respectively, see Figure 3A and Figure 3B. Finally, F_{y} and F_{el} represent the out-of-plane (from the supporting rod) and the in-plane forces. The radius of the microstructures is denoted by R , their out-of-plane distance is denoted by y, and their inter-axis distance is denoted by I_0. Additionally for the conical shape, α and β are the angles of the top and bottom cone, respectively. Here, the angular displacement of the microstructures is not considered for simplicity.

The single-pair interaction is assumed to be independent from the neighbouring structures as well as linearly elastic, as typically done in the classical multi-asperity contact formalism [3]. Whilst this approximation properly holds for dilute systems, a condition met when a low density of micro-structures (dilution) occurs on both the patterned surfaces, the other limiting condition, i.e. the dense system (a single structure interacts with the multiple surrounding ones), can be approximately modelled by assuming a constant in-plane lattice distance between the contacting microstructures during the interaction. Thus, for the dense system, the equivalent microstructure bending stiffness (Figure 3C(bottom)) is assumed infinite (the surrounding structures do impede in-plane displacements), whereas, for the dilute system, the bending stiffness of the microstructures and its contribution to the in-plane displacements need to be considered.

Finally, the contact case of elongated microstructures might show the occurrence of elastic instability, locally hindering the setting of interlocking. In Figure 3C(top) the sticking-induced buckling is schematically represented. As shown in the following, this phenomenon is mainly associated with smooth-ending microstructures and dense packing, and is taken into account in our model.

4.1 Microgeometry with spherical tip

The first interlocking microgeometry analysed here is the sphere, due to its smallest set of geometrical descriptors, as well as due to the availability of an analytical description of the local (point) contact. In particular, we make use of the Johnson, Kendall and Roberts contact theory [4]. As known, for the point contact the interaction force and the penetration are related by the following system of equations:

$$F_{\mathrm{n}} = \frac{4E^{*}a^{3}}{3R^{*}} - \sqrt{8\pi\Delta\gamma E^{*}a^{3}} \tag{1}$$

$$\delta = \frac{a^{2}}{R^{*}} - \sqrt{\frac{2\pi\Delta\gamma a}{E^{*}}}, \tag{2}$$

where $R^{*} = R_{\mathbf{1}}R_{\mathbf{2}}/(R_{\mathbf{1}} + R_{\mathbf{2}})$ is the equivalent contact radius, E^{*} is the reduced elastic modulus $E^{*-1} = (1-\nu_1^2)\,E_1^{-1} + (1-\nu_2^2)\,E_2^{-1}$, whereas $\Delta\gamma$ is the true work of adhesion. The subscript 1 (2) is for the bottom (top) contacting solid.

4.1.1 Dense system

When the spheres are densely packed their inter-axis distance is constant due to the multiple interactions with the surrounding spheres ($I = I_0$); the penetration δ is:

$$\delta = 2R - \sqrt{y^{2} + I_0^{2}}, \tag{3}$$

whereas the tangential force F_{t} is:

$$F_{\mathrm{t}} = \pm\mu F_{\mathrm{n}}, \tag{4}$$

where the sign depends on the direction of slipping we are considering: The + (-) sign is for approach (detach) direction. The out-of-plane force F_{y} can thus be obtained by solving Equations (1) to (4) and the out-of-plane momentum with respect to the out-of-plane distance y leads to:

$$F_{\mathrm{y}} = F_{\mathrm{n}}\frac{y}{\sqrt{y^{2}+I_0^{2}}} - F_{\mathrm{t}}\frac{I_0}{\sqrt{y^{2}+I_0^{2}}}. \tag{5}$$

Upon integration over y, the work of mechanical adhesion for the single sphere W_{sing} as a function of the in-plane distance I_0 can be calculated:

$$W_{\mathrm{sing}}(I_0) = -\int_{y_{\mathrm{F.C.D.}}}^{+\infty} dy\ F_{\mathrm{y}}(y, I_0), \tag{6}$$

where $y_{\mathrm{F.C.D.}}$ is the first contact distance, i.e. the out-of-plane distance when the first contact between spheres happens ($F_{\mathrm{y}} = 0$ when $y \leq y_{\mathrm{F.C.D.}}$). It is observed that when switching off the local adhesion $\Delta\gamma$ in Equations (1) and (2), only negligible differences occur in F_{y} and, thus, in $W_{\mathrm{sing}}(I_0)$; this is clearly shown in Figure S4A for the representative case of a single pair of interlocked spheres (having the same geometry as in the experimental panel in Figure 8 of the main manuscript, using the "dense" model at an interaxis distance equal to the radius).

Therefore, in the results reported in the main manuscript, the local adhesion $\Delta\gamma$ is neglected. This is a valid assumption for all the range of parameters investigated in this study. Note that Equations (1) to (6) are not strictly applicable for the approach case, due to the fewer interactions during this stage and to the occurrence of general elastic instability, which has to be modelled as a separate phenomenon. This global buckling during approach can be reduced in dilute systems, as occurs in biological surfaces.

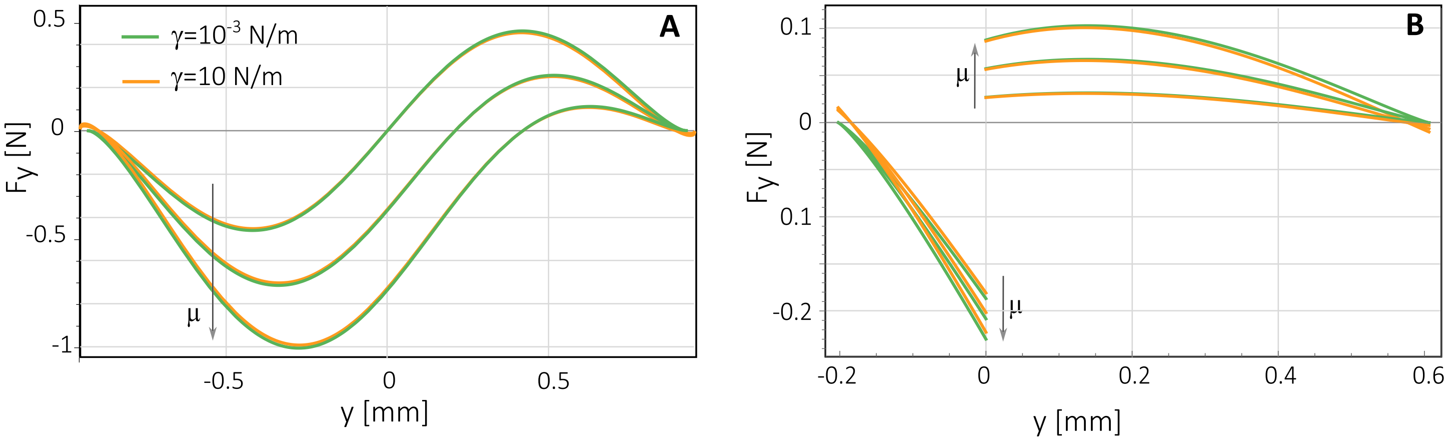

Figure S4: *Theoretical results.* A, Detachment force F_y as predicted by theory at various friction coefficient (μ) values, from top to bottom: μ=0, 0.2, 0.4 and two surface energies values: 10^{-3} N/m (equivalent to an adhesionless interaction) and 10 N/m. The soft sphere has an equivalent elastic modulus E^*=4 MPa, and radius R^*=0.7mm at interaxis distance I_0=1mm, predicted using the dense model. B, Detachment force F_y as predicted by theory at various friction coefficient (μ) values, from top to bottom : μ=0, 0.2, 0.4 and two surface energies values: 10^{-3} N/m (equivalent to an adhesionless interaction) and 10 N/m. The soft double cone has an equivalent elastic modulus E^*=4 MPa, and radius R^*=0.7mm at interaxis distance I_0=1mm, predicted using the dense model assumption.

4.1.2 Dilute system

As previously stated, modelling this system also requires introducing in-plane displacements, i.e. a finite supporting rod stiffness. Indeed, the latter has a key role in determining the normal force acting at the local contact. We assume a linear elastic response of the supporting rod, such that the in-plane force is proportional to the in-plane displacement of the interlocker, with relation:

$$F_{\mathrm{el}} = k(\sqrt{(R_1 + R_2 - \delta)^2 - y^2} - I_0), \tag{7}$$

where k is the equivalent lateral stiffness of the supporting rod, and R_1 and R_2 are the radii of the contacting spheres. The indentation δ is not given explicitly as a function of the variable y since it can not be derived from geometrical considerations. To determine δ, both the in-plane momentum and the out-of-plane momentum equations are needed:

$$F_{\mathrm{el}} = F_{\mathrm{n}} \frac{\sqrt{(R_1 + R_2 - \delta)^2 - y^2}}{R_1 + R_2 - \delta} - F_{\mathrm{t}} \frac{y}{R_1 + R_2 - \delta} \tag{8}$$

$$F_{\mathrm{y}} = F_{\mathrm{n}} \frac{y}{R_1 + R_2 - \delta} + F_{\mathrm{t}} \sqrt{1 - \left(\frac{y}{R_1 + R_2 - \delta}\right)^2}. \tag{9}$$

Neglecting the true work of adhesion in Equations (1) and (2), and solving the system of equations Equations (1), (2), (4) and (7) to (9), then Equation (8) can be reduced to a single equation relating the contact radius a to the free variable y:

$$k_1 a^3 \left(\frac{\sqrt{(R_1 + R_2 - k_3 a^2)^2 - y^2}}{R_1 + R_2 - k_3 a^2} - \mu \frac{y}{R_1 + R_2 - k_3 a^2}\right) = k(\sqrt{(R_1 + R_2 - k_3 a^2) - y^2} - I_0), \tag{10}$$

where $k_1 = 4E^*/3R^*$ and $k_3 = 1/R^*$. Once $a\,(y)$ is determined from Equation (10), δ can be computed. In order to obtain the work of adhesion for the single sphere we use Equation (6). Note that:

$$[F_{\mathrm{y}}(y)]_{approach} = -\,[F_{\mathrm{y}}(-y)]_{detach} \tag{11}$$

for the spherical model as can be seen in Figure S5 when comparing the approach and recede motion graphs, since the problem is symmetrical for the spheres interaction case. Figure S5 shows numerical results for the spherical tip contact mechanics for both dense (left side) and dilute (right side) systems.

4.1.3 Role of jamming and buckling in the approach motion

It is observed that a critical y_{crit} exists such that the single microgeometry contact occurs under jamming (stick contact), with $y_{\mathrm{crit}} = \frac{I_0}{\mu}$. Indeed, for $y > y_{\mathrm{crit}}$ the model shows a non-physical solution with a negative normal force F_{n}. During jamming, increasing the normal local contact force determines an increase of the local tangential force, but without initiating the sliding. Thus, the interlocker will undergo elastic or irreversible buckling depending on the structural properties of the supporting rod. A critical interaxis distance I_{crit} can be defined as the distance at which contacting structures will not slide when they have $I_0 < I_{\mathrm{crit}}$. I_{crit} is obtained by equating y_{crit} to the y value at which the first contact happens during approach, $y_{\mathrm{F.C.A.}} = \sqrt{(R_1 + R_2)^2 - I_0^2}$. Thus $I_{\mathrm{crit}} = \mu(R_1+R_2)/\sqrt{1+\mu^2}$. In the case $I_0 < I_{\mathrm{crit}}$ the supporting rod will first deform such as an elastic rod, until the critical buckling threshold is reached:

$$F_{\mathrm{y,crit}} \approx \frac{\pi^2 E_{\mathrm{rod}} J_{\mathrm{rod}}}{l_{\mathrm{rod}}^2}, \tag{12}$$

where J_{rod} is the geometric moment of inertia of the supporting rod section, l_{rod} the length of the supporting rod, and E_{rod} is the elastic modulus of the rod material. After the buckling deformation, the out-of-plane load $F_{\mathrm{y}} \approx F_{\mathrm{y,crit}}$ during subsequent approach motion, as schematically shown in Figure 7A.2 of the main manuscript. As a result, the jammed microsphere does not interlock, decreasing the amount of effective work of adhesion, as described in Supplementary Section 6.

4.2 Microgeometry with double conical tip

The largest disadvantage of spherical interlockers is the lack of geometrical parameters to be changed in order to optimise the system depending on the specific application. In this respect, a more optimisable shape is the double conical microgeometry, whose tip angle can be designed to avoid either sticking and buckling phenomena during the approaching motion, whilst the docking angle (see β in Figure 3A of the main manuscript) can be chosen to maximise the pull-off force without exceeding the admissible tensile stress of the material.

The first problem to assess is finding a relation between the geometrical parameters involved in the contact (contact length l and penetration δ) and the relative position of two cones, identified by their in-plane interaxis distance I and out-of-plane distance y. Given the discontinuous nature of a double cone geometry, such a relation is also discontinuous and has two different branches for interlocked and non-interlocked position. This discontinuity has been also observed experimentally in Figure S7A and B by measuring the interaction force between a double conical single pair in the attaching phase for two different friction coefficients, using the same setup described in Figure 8C. Furthermore, the non-interlocked branch shows two more branches depending on whether the cone tips are contacting the cone surfaces, see the schematic contact representation at different penetration steps in Figure 3A of the manuscript). $\delta(y, I)$ and $l(y, I)$ are:

$$\delta(y,I) = \begin{cases} (2R - I)\cos\alpha - y\sin\alpha & \text{for } y \geq 0 \\ (2R - I)\cos\beta + y\sin\beta & \text{for } y < 0 \end{cases} \tag{13a}$$

$$l(y,I) = \begin{cases} \frac{(2R+I)\cos\alpha - y\sin\alpha}{\sin 2\alpha} & \text{for } y \geq 0, I < R \\ \frac{(2R-I)\cos\beta + y\sin\beta}{\sin(\alpha+\beta)} & \text{for } y \geq 0, I \geq R \\ \frac{(2R-I)\cos\alpha - y\sin\alpha}{\sin(\alpha+\beta)} & \text{for } y < 0. \end{cases} \tag{13b}$$

In analogy with the spherical case and limiting the investigation to the dense system, it is possible to neglect the influence of surface energy, since it has a minor impact in the effective work of adhesion. In order to justify this assumption an exact formulation for the adhesive contact between parallel cylinders based on JKR adhesive theory has been used, which relates the normal force per unit length of contact f_{n} to the penetration δ [5–7]:

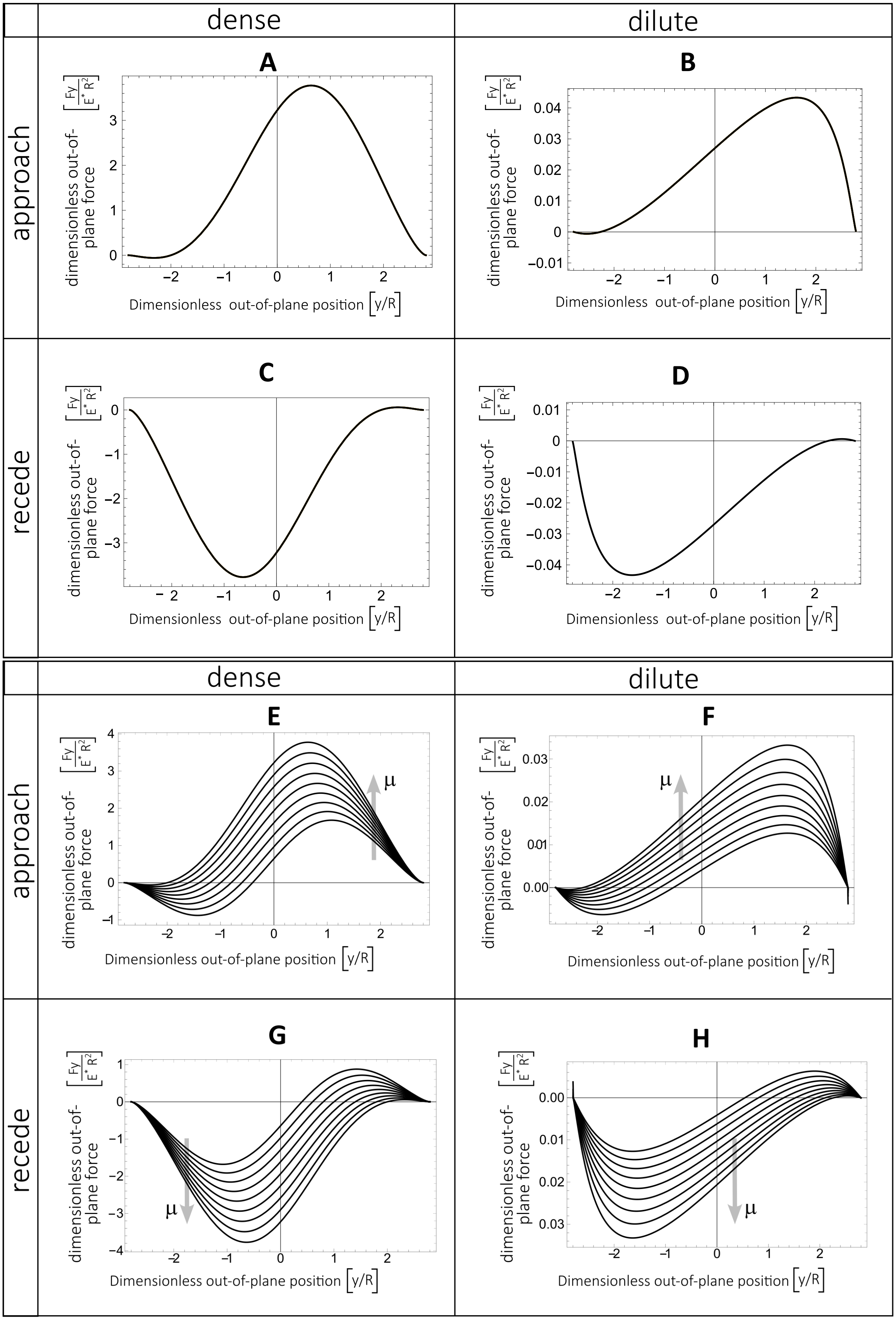

Figure S5: *Theoretical results.* A, B, C, D, Normalized single pair interaction model for interlocking spheres (radius R=0.7 mm; $\mu = 0.7$; $I_{\mathbf{0}} = 1$ mm). E, F, G, H, Normalized single pair interaction model for interlocking spheres parameterised with respect to the friction coefficient (varying between 0.14 and 0.7, with step 0.07). Note that the interaction force increases with it.

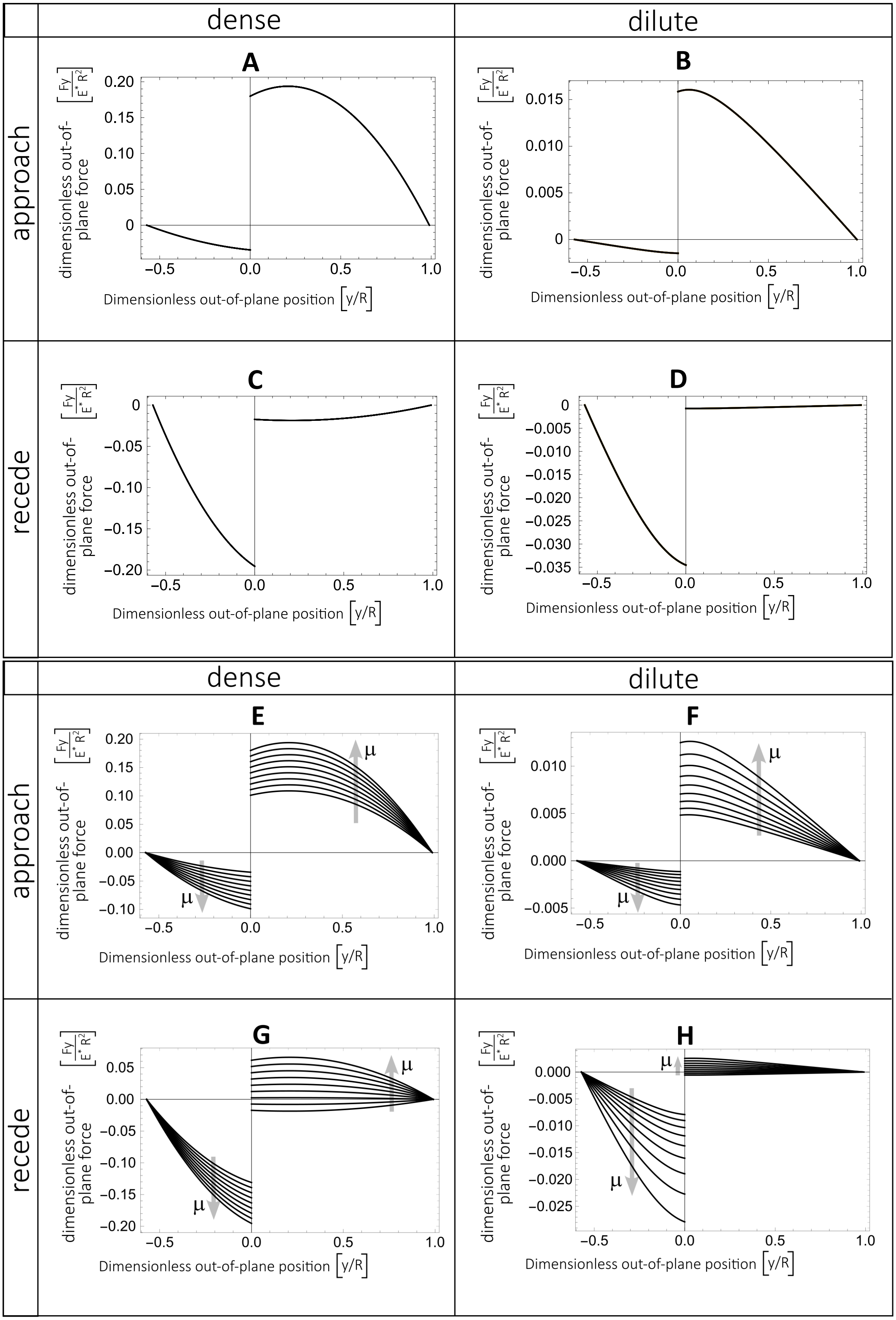

Figure S6: *Theoretical results.* A, B, C, D, Normalized single pair interaction model for interlocking double cones (radius R=0.7 mm; $\mu = 0.7$; $I_{\mathbf{0}} = 1$ mm). E, F, G, H, Normalized single pair interaction model for interlocking double cones parameterised with respect to the friction coefficient (varying between 0.14 and 0.7, with step of 0.07). Note that the interaction force increases with it.

$$f_{\mathrm{n}} = \frac{\pi E^{*} a^{2}}{4R} - (2E^{*}\pi a \Delta\gamma)^{1/2} \tag{14a}$$

$$\delta = \frac{1}{\pi E^{*}}\left(\frac{\pi E^{*} a^{2}}{4R} + 2log(2)F_{\mathrm{n}} - 2log(a)F_{\mathrm{n}} + 2log(d)F_{\mathrm{n}}\right). \tag{14b}$$

It is worth noticing that these relations depend on a datum point d which in this context has been assumed equal to the contact length l. For any value of y Equation (13) provide a value for δ and l. Then Equation (14) have been numerically integrated along the line of contact, since they depend on the local radius of the contacting bodies. The resulting F_{n} and F_{t} have been projected in the out-of-plane direction to finally give F_{y} as a function of y, see Figure S4B. In particular, similarly to the case of spherical tips (Figure S4A), only negligible differences occur in F_{y} when adhesion is included or not in the calculations. Therefore, the case of two identical interacting cones (with parallel axis) is hereinafter approximated to a cylindrical adhesionless contact, with varying contact radii along the contact line. In addition, Winkler foundation is adopted to further simplify the theory. For a cylindrical contact, in this simplified case the relation between normal force, penetration and contact length is linear [4]:

$$F_{\mathrm{n}} = \frac{\pi}{4} E^{*} \delta(y, I) l(y, I). \tag{15}$$

In the equation above, the local contact radius is not explicitly included, thus the equation can be easily used for cone-cone contact. We will make use of the same friction equation used for spheres in Equation (4), using the same sign convention: + (-) is for the approaching (detaching) phase.

4.2.1 Dense system

When the structures are densely packed together, their interaxis distance is constant, in analogy with what has been reported for the case of spherical structures. Hence, I in Equation (13) is constant, $I = I_0$. The out-of-plane momentum requires:

$$F_{\mathrm{y}} = \begin{cases} \frac{\pi}{4} E^{*} \delta(y, I) l(y, I) \left(\sin\alpha \pm \mu \cos\alpha\right) & \text{for } y \geq 0 \\ \frac{\pi}{4} E^{*} \delta(y, I) l(y, I) \left(-\sin\beta \pm \mu \cos\beta\right) & \text{for } y < 0. \end{cases} \tag{16}$$

Equation (6) is used to calculate the detachment work for the single cone. In this case the geometry is described by two different angles (α and β), so there will be a noticeable difference between the approaching and the receding motion, and the interaction is not symmetric as for the case of spheres (see Figure S6)

4.2.2 Dilute system

As in the case of dilute spherical interlockes (Supplementary Section 4.1.2), here the supporting rod compliance is finite. The in-plane momentum equation is needed to determine the actual in-plane interaxis $I(y)$:

$$k(I - I_0) = \begin{cases} \frac{\pi}{4} E^{*} \delta(y, I) l(y, I) \left(\cos\alpha - \pm\mu \sin\alpha\right) & \text{for } y \geq 0 \\ 4E^{*} \delta(y, I) l(y, I) \left(\cos\alpha \pm \mu \sin\alpha\right) & \text{for } y < 0 \end{cases} \tag{17}$$

where again y is the free variable. Upon finding $I(y)$, the latter can be substituted in Equations (13) and (16) to obtain the explicit relation between the vertical force F_{y} and vertical position y. Again, Equation (6) is used to calculate the detachment work for the single cone. Figure S6 shows numerical results for the double-cone tip contact mechanics for both dense (left side) and dilute (right side) systems.

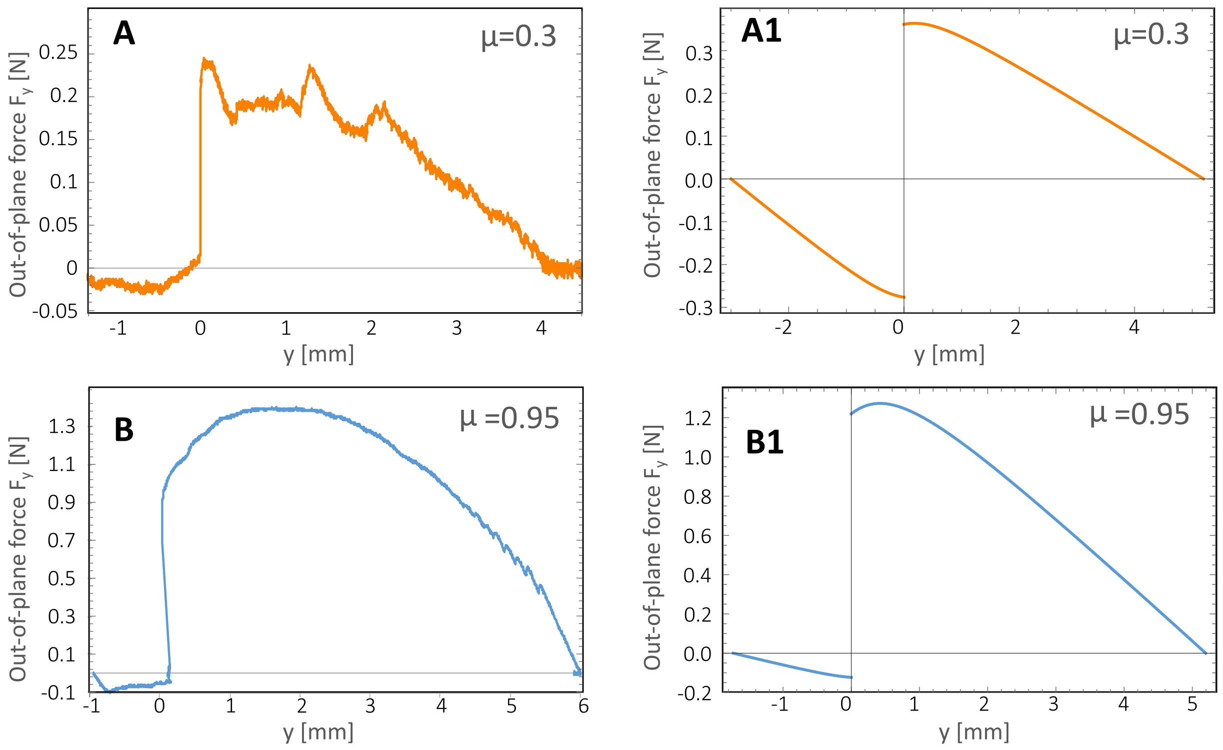

Figure S7: *Experimental and theoretical results.* Out of plane force (F_y) as a function of the out-of-plane distance between the double conical single-pair tips. A,B, Experimental results, A1,B1, numerical predictions. The results are for the attaching phase, at different values of friction coefficient (representative of the dry and dusty environment). The cones have radius R = 3mm.

5 Deterministic, quasi-random and random distribution of micro-structures

The probability density function of the distance between mating micro-structures $p(I_0)$ needs to be determined in order to predict the overall (or effective) work of adhesion (see Supplementary Section 6). In this work, it is always assumed that the relative angular position of the two interacting surfaces is random, with uniform distribution. This unlocks the opportunity to predict the effective mechanical work of adhesion for real applications under realistic interaction conditions. Furthermore, the position of the single structure is modelled with either a *i* deterministic lattice positioning, *ii* random with uniform distribution of positioning, or *iii* quasi-random positioning, over a finite circular domain (thus macroscopically isotropic), as reported in Figure 6C1 to C3 in the manuscript. Again, we stress here that the finiteness of the interaction domain is needed in order to model interlocking systems for the most general application, where e.g. the size of the single interlocker might not be much smaller than the apparent contact area representative size.

Let us consider two independent position probability distributions of points *(a)* and *(b)* over a limited domain A, respectively $P_{\mathrm{a}}(\mathbf{x})$ and $P_{\mathrm{b}}(\mathbf{x})$. Thus, the probability distribution of the distance I_0 between the two points *(a)* and *(b)* is given by:

$$p(I_0) = \int_A d^2x \; P_{\mathrm{a}}(\mathbf{x}) \left[\oint dl' \; P_{\mathrm{b}}(\mathbf{x}') \right], \tag{18}$$

where the line integral is computed over a circle of radius I_0 centred in $\mathbf{x}$. It is observed that, because of the finite domain, the inner integral will not be computed over a closed curve for some values of $\mathbf{x}$.

5.1 Interaction of uniformly random surfaces

It is assumed that our contact domain is a circle of radius R_{max} centered in the origin of the plane. The case of uniformly random distribution can be expressed in polar coordinates (r, θ):

$$P(r, \theta) = \begin{cases} \frac{1}{\pi R^2_{\mathrm{max}}}, & \text{for } 0 \le r \le R_{\mathrm{max}} \\ 0 & \text{otherwise} \end{cases}. \tag{19}$$

The inner closed integral in Equation (18) is solved by geometrical considerations, multiplying the perimeter of a circle of radius I_0 that lies inside the domain A to P_{b}:

$$\oint P_{\mathrm{b}}(\mathbf{x}')dl = \begin{cases} \frac{2I_0}{R^2_{\mathrm{max}}}, & \text{for } r \le R_{\mathrm{max}} - I_0 \\ \frac{2I_0}{\pi R^2_{\mathrm{max}}} \left(\pi - \arccos\left(\frac{R^2_{\mathrm{max}} - r^2 - I_0^2}{2rI_0} \right) \right) + \frac{2I_0}{R^2_{\mathrm{max}}} & \text{for } R_{\mathrm{max}} - I_0 \le r \le R_{\mathrm{max}} \end{cases}. \tag{20}$$

Equation (20) is thus multiplied by P_{a} and integrated over the circular domain splitting the integral when the branch of the equation changes, getting the final distribution of distances between the two surfaces. This integral has an analytical solution for both branches of the equation but, since we are dealing with distances much smaller than the overall dimension of the contacting plane, the first branch can be used to get the probability distribution of distances:

$$p(I_0) \approx \frac{2I_0}{R^2_{\mathrm{max}}}. \tag{21}$$

which becomes:

$$p(I_0) = \begin{cases} \frac{2I_0}{R^2_{\mathrm{max}}}, & \text{for } I_{0\,\mathrm{min}} < I_0 \le I_{0\,\mathrm{max}} \\ \frac{I^2_{0\,\mathrm{min}}}{R^2_{\mathrm{max}}} \delta(I_0 - I_{0\,\mathrm{min}}) & \text{for } I_0 = I_{0\,\mathrm{min}} \\ 0 & \text{Otherwise} \end{cases}, \tag{22}$$

where $\delta(I_0 - I_{0\,\mathrm{min}})$ is a Dirac delta function that accounts for the structures that are forced to be at a minimum distance imposed by the contact with the supporting rod, as presented in Figure 6B in the main manuscript, and $I_{0\,\mathrm{max}}$ is the tangency limit of the structures.

5.2 Interaction of deterministic or quasi-random surfaces

It is very interesting to investigate the dependence of the probability distribution of distance between mating microgeometries ($p(I_0)$) for a different nature, say deterministic or quasi-random, of surface patterning. To do so, Monte Carlo simulations have been executed in order to generate replications of quasi-random patterns (obtained by random placing points with respect to an ordered lattice, see Figure 6CY). The random placing is modelled with a Gaussian distribution, with standard deviation smaller that the lattice distance of the deterministic pattern) or deterministic patterns (see Figure 6CX, which also serves as deterministic initial lattice for the quasi-random pattern), and to determine their distance probability distributions, as reported in Figure 6C of the main manuscript. It is clearly observed that, even by changing the nature of the surface pattern, the distance probability distributions are mostly quantitatively unaffected. This is trivial for the deterministic and quasi-random pattern, since the distance distribution of the latter can be obtained by convolution of the distribution of the former with a Gaussian: since the Gaussian standard deviation is smaller that the lattice distance in the deterministic pattern, the convolution is equivalent to a convolution with the Dirac delta, returning the same distribution of the deterministic pattern, see Figure 6CX and CY.

5.3 Role of pattern substrate deformation on the distance probability distribution

In this work we introduce the modulation of mechanical adhesion by actively controlling the probability density functions of the microstructure distance. In particular, a facile approach to modulate adhesion in a controllable and quick way is to realise surface patterning on the top of soft substrates. Thus, by inflating the substrate where the microstructures are located, the initially flat surface can undergo an increase in surface area which intimately affects the areal density of structures, hence reducing the number of interactions and the overall effective work of engagement and adhesion. As a consequence, controlling the substrate deformation, e.g. with inflation here, can be adopted to fabricate switchable adhesives, to cite one application. To this purpose, the strategy would be to inflate a surface in the approaching phase, thus reducing the effective engagement work (needed for attachment), and deflating it to secure the interlocking (thus increasing the effective work of adhesion). In order to detach again, an inflation does reduce again the amount of work of adhesion, allowing to easily disengage the interfaces. In the following, the modelling to design switchable mechanical adhesives is reported.
Consider a circular elastic membrane or radius R_{max}, as shown in Figure 9C in the main manuscript. Upon inflation (i.e., upon application of a constant pressure field), the membrane deforms accordingly to the classical theory of plates [8], which relates the deformation normal to the plate $w(\mathbf{x})$ to the applied pressure on the surface p:

$$\Delta^2 w = \frac{p(\mathbf{x})}{D}, \tag{23}$$

where D is the stiffness of the plate, given by $D = \frac{Es^3}{12(1-\nu^2)}$, E the Young modulus, ν the Poisson coefficient and s the thickness. Assuming the simply supported boundary condition, with uniform pressure field, the out-of-plane displacement field reads:

$$w(r) = \frac{pr^4}{64D} - \left(\frac{pR_{\text{max}}^2}{32D}\frac{3+\nu}{1+\nu}\right) r^2 + \frac{pR_{\text{max}}^4}{64D}\frac{5+\nu}{1+\nu}, \tag{24}$$

where r is the distance from the center of the plate. Upon inflation, the apparent surface (C) linking the interlocker tips does increase even more than the substrate area increase, as schematically shown in the inset of Figure 9C of the main manuscript. In particular, the taller the interlockers, the larger the area increase. In the following, it is shown how to compute the aforementioned area increase.
To simplify the theory, Equation (24) is approximated first to a paraboloid, built to show the same displacements in $r = 0$ and $r = R_{\text{max}}$. The surface C is then described by the following implicit relation:

$$C := \begin{cases} r(t) = t - \frac{w'(t)h}{\sqrt{1+w'^2(t)}} \\ w_2(t) = w(t) + \frac{h}{\sqrt{1+w'^2(t)}}, \end{cases} \tag{25}$$

$w'(t)$ is the first derivative of $w(t)$, h is the offset value (the height of interlocking structures) and t is the free parameter in the domain $[-R_{\max}, R_{\max}]$. The area A_1 of this surface can be computed with the following integral:

$$A_1 = 2\pi \int_0^{R_{\max}} dt\ r(t)\sqrt{r'(t)^2 + w_2'(t)^2}. \tag{26}$$

It is finally possible to compute the ratio between the new area value and the initial undeformed one $q = \frac{A_1}{\pi R_{\max}^2}$. The change in the distance distribution between interlockers is linked to the new equivalent radius of the inflated surface, calculated as:

$$R_{\text{eq}} = R_{\max}\sqrt{q}, \tag{27}$$

that is the only parameter needed to define the new distance probability distribution altered by inflation as seen in Equation (21).

6 Cohesive Zone Model and Effective work of mechanical adhesion

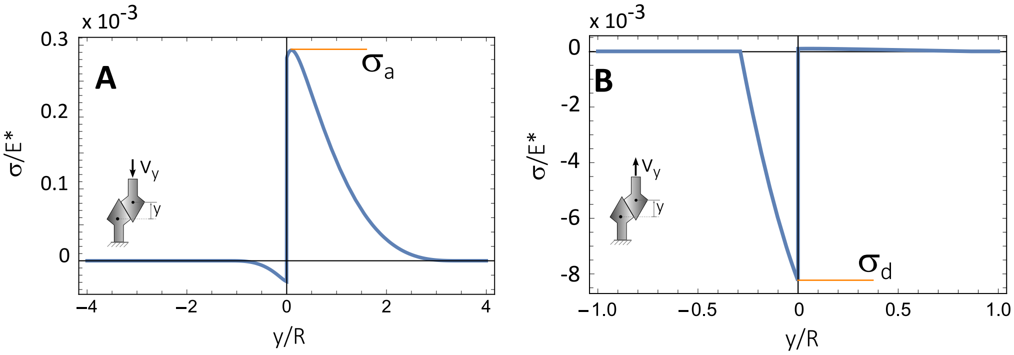

Figure S8: *Theoretical results.* Equation of state relating averaged normalized pressure to normalized out-of-plane displacement for conical structures. The geometrical descriptors and material properties are the same as used in the experimental section in Figure 8 in the main manuscript. A, attachment motion: σ_{a} is the attachment limit pressure needed for full engagement of the surfaces when they are approaching each other (V_{y} is the out-of-plane velocity, directed downwards during approaching). B, detachment motion: σ_{d} is the detachment limit pressure needed for full disengagement of the surfaces when they are moving away from each other (V_{y} is the out-of-plane velocity, directed upwards during receding)

The effective work of adhesion can be obtained as a result of the following integral:

$$\gamma_{\text{eff}} = \frac{n_{\text{a}}\, n_{\text{b}}}{\pi\, R_{\max}^2} \int_0^{I_{0\ max}} p(I_0)\, W_{\text{sing}}(I_0)\, dI_0, \tag{28}$$

given by the product of Equation (21) and Equation (6), where n_{a} and n_{b} are the number of structures on the two surfaces in contact. It is stressed that, depending on the number of structures on the surfaces, rather the densely packed model or the non densely packed model should be used. For the conical case, $I_{0\ \min}$ is the supporting rod limit as presented in Figure 9C in the main manuscript: The structures closer than this limit are considered through the Dirac delta as discussed in (Supplementary) Section 5 and shown in Figure 6B in the manuscript. In other terms, interlocking occurs together with the sliding contact between the supporting rod of the neighbouring microstructures. For the spherical case, $I_{0\ \min}$ is the buckling/jamming limit, so that for distances lower than this limit interlocking is not physically obtainable, in this case the structures closer than this limiting threshold are not considered through the

Dirac delta function, since it is assumed they do not slide during the interaction. Analogously, averaging the force of the single interaction in the same distance range $[0, I_{0\,\mathrm{max}}]$ and dividing by the apparent contact area we can extract the equation of state of the interaction, linking the pressure during the attachment process (σ_a in Figure S8A) and the pressure during the detachment process (σ_d in Figure S8B) to the out-of-plane distance:

$$\sigma(y) = \frac{n_\mathrm{a}\, n_\mathrm{b}}{\pi\, R_\mathrm{max}^2} \int_0^{I_{0\,max}} p(I_0)\; F_\mathrm{y}(y, I_0)\, dI_0. \tag{29}$$

The predicted effective adhesion values slightly overestimate the detachment work obtained by experiments, see Figure 8. This mismatch is due to the model approximations, including the adoption of the Hertzian contact, up to the Coulomb friction assumption and to the approximation of the distance distribution. The elastic energy stored in the buckled structures does also affect the work of adhesion by reducing it. Overall, there is fairly good agreement between theory and experimental results.

References

[1] H. Hertz, *J Reine Angew, Math* **1881**, *92* 156.

[2] K. L. Johnson, K. Kendall, A. Roberts, *Proceedings of the royal society of London. A. mathematical and physical sciences* **1971**, *324*, 1558 301.

[3] J. Greenwood, J. Williamson, *Proceedings of the Royal Society of London (A)* **1966**, *295* 300.

[4] K. L. Johnson, *Contact mechanics*, Cambridge university press, **1987**.

[5] M. K. Chaudhury, T. Weaver, C. Hui, E. Kramer, *Journal of Applied Physics* **1996**, *80*, 1 30.

[6] M. Barquins, *The Journal of Adhesion* **1988**, *26*, 1 1.

[7] J.-J. Wu, *Tribology Letters* **2019**, *67*, 1 19.

[8] S. Timoshenko, S. Woinowsky-Krieger, et al., *Theory of plates and shells*, volume 2, McGraw-hill New York, **1959**.
